# Data-driven serum cytokine and chemokine phenotypes are associated with MRI-defined disc regression and persistent radicular pain in lumbar disc herniation

**DOI:** 10.3389/fimmu.2026.1928963

**Published:** 2026-09-03

**Authors:** Wei Shen, Ke Yang, Yang Liu, Tian Hao, Haiyu Zhou, Hua Huang

**Affiliations:** 1 Beijing Anzhen Nanchong Hospital, Capital Medical University & Nanchong Central Hospital, Nanchong, China; 2 Chengdu Xinhua Hospital Affiliated to North Sichuan Medical College, Chengdu, China; 3 The Second Hospital & Clinical Medical School, Lanzhou University, Lanzhou, China

**Keywords:** CCL2, chemokines, cytokines, IL-6, IL-8, lumbar disc herniation, MMP-9, MRI-defined disc regression

## Abstract

**Background:**

Inflammation after lumbar disc herniation (LDH) may contribute to radicular pain while also participating in resorption of herniated disc material. Whether circulating immune profiles are associated with these endpoint-specific recovery patterns remains uncertain.

**Methods:**

This single-center observational cohort included patients with symptomatic LDH treated between 1 January 2023 and 30 November 2025. Stored baseline serum IL-6, IL-8/CXCL8, CCL2/MCP-1, and MMP-9 were log-transformed, standardized, and entered into outcome-blinded Gaussian mixture modeling. MRI-defined regression (>=50% volume reduction during nonoperative follow-up) and persistent radicular pain (recorded follow-up leg-pain VAS >=3) were analyzed in endpoint-specific cohorts. Selection weighting, early-surgery-as-failure, classification-certainty, storage-adjusted, continuous-outcome, and penalized-model sensitivity analyses were performed.

**Results:**

Among 146 patients with complete four-marker data, the BIC-preferred EII three-class solution identified low immune activation (n=46), cytokine-dominant (n=32), and chemokine/MMP-dominant (n=68) phenotypes (Delta BIC 7.29 versus the next-best model). In the paired-MRI cohort (N = 117), regression occurred in 52.6%, 40.9%, and 71.9%, respectively. The chemokine/MMP-dominant phenotype was associated with MRI regression in the primary adjusted model (OR 2.99, 95% CI 1.11-8.08; P = 0.031; global P = 0.019), and adjusted continuous volume reduction was 9.88 percentage points greater than in the low-activation group (95% CI 0.08-19.68; P = 0.048). Several sensitivity analyses retained the direction but widened uncertainty, including nonlinear-age, early-surgery, and posterior-certainty analyses. In the persistent-pain cohort (N = 123), the categorical phenotype association was heterogeneous but class-specific estimates were imprecise; the continuous IL-6/IL-8 cytokine axis showed the clearest association with persistent pain (OR per 1-SD 2.38, 95% CI 1.34-4.21; P = 0.003).

**Conclusion:**

Baseline serum immune profiles were associated with endpoint-specific structural and pain outcomes in LDH. The chemokine/MMP-dominant phenotype showed the clearest association with MRI-defined regression, whereas persistent pain was more consistently related to a continuous cytokine axis. These observational findings are hypothesis-generating and require prospective multicenter and tissue-level validation before clinical use.

## 1 Introduction

Lumbar disc herniation (LDH) is commonly managed as a mechanical disorder, yet the clinical course is often more complex than the size of the herniated fragment alone would suggest. Many patients improve with nonoperative care, whereas others have persistent radicular pain or require surgery despite apparently comparable baseline imaging. This clinical heterogeneity is one reason why guideline-level summaries emphasize both natural recovery and careful selection for surgery rather than relying on morphology alone (1–3). More broadly, low back pain and sciatica remain major contributors to disability, and persistent pain is shaped by biological and non-biological mechanisms that are not fully captured by structural imaging (4).

Spontaneous regression of herniated disc material is now a well-recognized phenomenon. Early radiological reports documented regression of herniated nucleus pulposus without surgery, and later systematic reviews showed that regression is particularly common in extruded or sequestered herniations (5–7). More recent reviews have reinforced that disc resorption is not a passive event, but a biologically active process involving exposure of nucleus pulposus to the epidural space, neovascularization, macrophage infiltration, phagocytic clearance, and extracellular-matrix degradation (8–10). Contrast-enhanced MRI studies also suggest that rim enhancement around herniated material may reflect vascularized inflammatory tissue and may relate to subsequent resorption (11, 12).

The same inflammatory setting can also generate pain. Disc cells and herniated disc tissue can produce proinflammatory cytokines and chemokines, and these mediators may sensitize nerve roots or amplify neuroimmune signaling (13, 14). Human disc tissue has been shown to produce monocyte chemoattractant protein-1 and interleukin-8, while experimental herniation models have demonstrated early production of TNF-alpha, IL-1beta, and MCP-1 after disc exposure (15, 16). In parallel, macrophage infiltration and matrix metalloproteinase activity have been implicated in the enzymatic and phagocytic breakdown of herniated material (17, 18). This creates an apparent immune paradox: inflammation may contribute to radicular pain, but selected inflammatory programs may also facilitate structural resorption.

Serum studies provide a clinically feasible window into this biology, although circulating markers are necessarily indirect. A prospective study of lumbar radicular pain found higher serum IL-6 and IL-8 among patients with persistent pain after disc herniation (19). A serum protein profiling study further suggested that patients with severe pain one year after disc herniation differed from recovered patients across multiple inflammatory proteins (20). Separately, serum cytokines and matrix metalloproteinases have been reported to vary across intervertebral disc disease phenotypes, supporting the plausibility of measurable systemic inflammatory correlates (21). These studies, however, largely focused on individual markers or pain outcomes and did not integrate structural disc regression and clinical pain persistence within a single immune-phenotyping framework.

Because there are no validated clinical cutoffs defining serum “resorption” or “pain-inflammatory” immune states in LDH, arbitrary thresholding is vulnerable to *post hoc* interpretation. A data-driven, outcome-blinded phenotype strategy is therefore more defensible when the aim is to identify immune-response patterns rather than test one biomarker in isolation. We hypothesized that baseline serum cytokine and chemokine profiles would identify clinically meaningful immune phenotypes, and that these phenotypes would show different endpoint-specific associations with MRI-defined disc regression and persistent radicular pain. Specifically, we examined whether a chemokine/MMP-dominant phenotype would align with disc resorption, and whether a cytokine-dominant inflammatory pattern would align with pain persistence.

## 2 Materials and methods

### 2.1 Study design and reporting

This was a single-center observational cohort study of patients with symptomatic LDH, using nested clinical, paired-MRI, and stored-serum ELISA cohorts. The study period was 1 January 2023 to 30 November 2025. The study evaluated baseline circulating immune profiles in relation to subsequent structural and clinical outcomes. Reporting was guided by the STROBE principles for observational studies (22). The study was not designed as a clinical prediction-model development study, and no prediction claims are made (23). The study was not prospectively registered, and no formal statistical analysis plan was registered before outcome ascertainment; therefore, inferential analyses beyond the outcome-blinded phenotype derivation are interpreted as exploratory.

### 2.2 Study population and nested cohorts

The source population comprised all eligible symptomatic LDH records represented in the institutional cohort during the study period. Eligibility required radicular leg pain clinically consistent with the symptomatic disc level and baseline lumbar MRI. Exclusions were spinal infection, tumor, fracture, inflammatory spondyloarthritis, previous surgery at the same level, cauda equina syndrome requiring emergency surgery, severe lumbar spinal stenosis as the dominant pathology, active malignancy, pregnancy, recent systemic infection, autoimmune disease requiring immunosuppressive therapy, chronic systemic corticosteroid or biologic immunomodulator use, poor-quality baseline MRI, or serum unsuitable for ELISA. Eligibility was established at the source-cohort level before serum, follow-up MRI, or clinical follow-up availability defined the nested analytic sets.

Nested cohorts were defined in parallel rather than as a single sequential pathway. Stored serum was available in 160 patients, 154 met assay-validity criteria, and 146 had complete positive values for the four primary phenotype markers. Paired MRI was available in 170 source patients and clinical follow-up in 230. The primary MRI analysis included 117 patients with phenotype assignment plus paired MRI, and the persistent-pain analysis included 123 with phenotype assignment plus the clinical endpoint. The MRI-clinical overlap comprised 104 patients; 96 had complete data for all exploratory composite components. Patients undergoing surgery before an evaluable follow-up MRI were excluded from the primary MRI endpoint but were included as unfavorable structural outcomes in a sensitivity analysis addressing informative surgical conversion.

### 2.3 Clinical and treatment variables

Baseline variables included age, sex, body mass index, smoking, cardiometabolic comorbidities, symptom duration, baseline VAS leg and back pain, ODI, neurological signs, target level, morphology, baseline herniation volume, nerve-root compression, Pfirrmann grade, Modic type, CRP, NLR, and treatment exposures before blood sampling. Post-baseline data available in the clinical cohort included epidural steroid injection within 6 months, total analgesic-use days, analgesic persistence, rehabilitation adherence, surgery within 6 months, and rescue visits. Longitudinal NSAID- and oral-steroid-specific dose trajectories were not captured in sufficient detail for treatment-specific causal analyses.

### 2.4 MRI assessment and reliability

Baseline and follow-up MRI examinations were evaluated at the symptomatic target level. The baseline-to-follow-up interval was recalculated directly from the recorded MRI dates rather than from admission date. The analytic dataset contained patient-level herniation volumes and a recorded volume-method field; in the primary MRI cohort, the recorded methods were ellipsoid approximation (59/117), semi-automated volumetry (33/117), or reader-consensus measurement (25/117). Percentage volume reduction was calculated from the paired baseline and follow-up volumes. MRI regression >=50% was used as a pragmatic dichotomous structural endpoint rather than as a universally validated LDH cutoff; >=70% regression was secondary, and adjusted continuous percentage volume reduction was analyzed to reduce dependence on dichotomization.

An archived inter-reader reliability summary was available for the MRI assessments. Agreement estimates ranged from 0.706 to 0.818 for nominal categorical ratings (kappa), from 0.804 to 0.876 for ordinal ratings (weighted kappa), and from 0.834 to 0.956 for volumetric measures (ICC). Ten of 117 primary MRI analyses were flagged for reader disagreement in the analytic dataset and were resolved by consensus. The archived records did not retain the double-read sample size, reader training or blinding metadata, exact ICC parameterization, an intra-reader repeat dataset, segmentation software/boundary protocol, handling rules for migrated or sequestered fragments, same-platform status, scanner field strength, sequence parameters, or slice thickness. Accordingly, the available data support inter-reader agreement but do not establish scanner/platform reproducibility or full technical reproducibility of the volumetric workflow. MRI measurement methods, disagreement records, and the archived reliability summary are reported in **Supplementary Table 13**.

### 2.5 Serum ELISA markers and assay validity

Stored baseline serum samples were available in 160 patients and were collected 0–3 days after admission. These specimens were residual serum remaining after routine clinical blood collection rather than blood drawn solely for research purposes, and no repeated freeze-thaw cycles occurred before ELISA testing. The four primary phenotype-input markers were IL-6, IL-8/CXCL8, CCL2/MCP-1, and MMP-9; MMP-3 was treated as a secondary marker. Assay validity assessment included assay-validity status, complete positive core-marker values, duplicate coefficient of variation, lower-limit-of-quantification/dilution flags, plate information, storage duration, and recent minor-inflammation flags. Because storage duration correlated with CCL2 and MMP-9, storage-adjusted residualized marker profiles were reclustered as a sensitivity analysis. Assay-validity, timing, handling, and storage characteristics are summarized in **Supplementary Table 2**.

### 2.6 Data-driven immune phenotype modeling

Immune phenotypes were derived using outcome-blinded Gaussian mixture modeling. Only standardized log-transformed IL-6, IL-8/CXCL8, CCL2/MCP-1, and MMP-9 values were used for primary clustering; outcomes and non-analytic source fields were excluded. Candidate covariance structures and class numbers G = 1–6 were compared by Bayesian information criterion (BIC). Solutions were also screened for minimum class size (>=10 patients and >=5% of the phenotype cohort), posterior assignment certainty, and subsample stability. The selected solution was implemented using the mclust framework (24). The BIC-preferred EII three-class solution was retained; its BIC was -1602.831, 7.285 points better than the next-best model. Neutral phenotype labels (low immune activation, cytokine-dominant, and chemokine/MMP-dominant) were used in outcome reporting. Sensitivity analyses excluded assignments with maximum posterior probability <0.80 and repeated clustering after storage adjustment and with MMP-3 added as a fifth marker.

### 2.7 Outcomes

The primary imaging endpoint was MRI-defined disc regression during nonoperative follow-up, operationalized as >=50% reduction in herniation volume. The primary clinical endpoint was persistent radicular pain, operationalized as a recorded follow-up leg-pain VAS >=3; this threshold was treated as an analytic definition of clinically relevant residual pain rather than a validated LDH-specific diagnostic cutoff. The clinical follow-up closest to the intended 6-month assessment was used (observed interval 106–294 days in the analytic pain cohort). Eleven pain-cohort patients underwent surgery within 6 months; surgery preceded the recorded clinical assessment in nine and followed it in two. Main analyses used the recorded clinical outcome, and a sensitivity analysis excluded all patients undergoing surgery within 6 months. Continuous VAS, VAS change, ODI, ODI change, and percentage volume reduction were analyzed as complementary outcomes.

### 2.8 Statistical analysis

Continuous variables were summarized as median [interquartile range], and categorical variables as n (%). Baseline characteristics were compared across phenotypes using Kruskal-Wallis, chi-square, Fisher exact, or Monte Carlo chi-square tests as appropriate. Effect sizes were summarized using maximum pairwise standardized mean difference for continuous variables and Cramer’s V for categorical variables. Baseline P values were descriptive and were not used as the sole basis for confounder selection. No multiplicity adjustment was applied; secondary and sensitivity P values are therefore interpreted as descriptive/exploratory, with emphasis on effect estimates, confidence intervals, and consistency of direction.

Primary association analyses used logistic regression with low immune activation as the reference group. The MRI model adjusted for age, sex, body mass index, morphology, baseline herniation volume, and the corrected baseline-to-follow-up MRI interval. The pain model adjusted for age, sex, body mass index, baseline VAS leg pain, motor weakness as a marker of neurological severity, and morphology. These low-dimensional sets represented clinically plausible confounding domains rather than selection based only on univariable phenotype P values. Standard logistic models report Wald confidence intervals and pairwise P values; global phenotype P values use likelihood-ratio tests. Complete model coefficients, events-per-parameter metrics, variance inflation factors, quadratic functional-form checks, influence diagnostics, Brier scores, and Hosmer-Lemeshow tests are reported in the supplement.

Sensitivity analyses included Firth penalized logistic regression, modified Poisson regression with robust sandwich standard errors for common binary outcomes (25). MRI analyses additionally used flexible follow-up-time modeling, an age-nonlinearity model, exclusion of lower-certainty phenotype assignments, early surgery treated as structural failure, staged and combined inverse-probability weighting, storage-adjusted phenotype reassignment, five-marker clustering, and influential-observation exclusion. Clinical analyses additionally used staged and combined inverse-probability-of-follow-up weighting, storage-adjusted and five-marker phenotype definitions, influential-observation exclusion, restriction to patients without surgery by 6 months, and best/worst-case endpoint attrition scenarios. Stabilized weights were truncated at the 1st and 99th percentiles, and balance was assessed using standardized mean differences. Continuous MRI, VAS, and ODI models used baseline outcome adjustment where applicable and HC3 robust standard errors; MRI follow-up time was modeled flexibly in the continuous structural model. The cytokine axis was the mean of z-scored log IL-6 and IL-8/CXCL8, and the remodeling axis was the mean of z-scored log CCL2/MCP-1 and MMP-9. Primary endpoint-specific models had no missing covariates, so multiple imputation would not have increased their model sample size; endpoint attrition was evaluated separately by weighting and extreme-case sensitivity analyses.

### 2.9 Ethics

The study protocol, including retrospective clinical/imaging data review and analysis of stored de-identified residual serum remaining after routine clinical blood collection for ELISA-based biomarker measurement, was approved by the committee.

## 3 Results

### 3.1 Cohort construction and baseline characteristics

**Figure 1:**
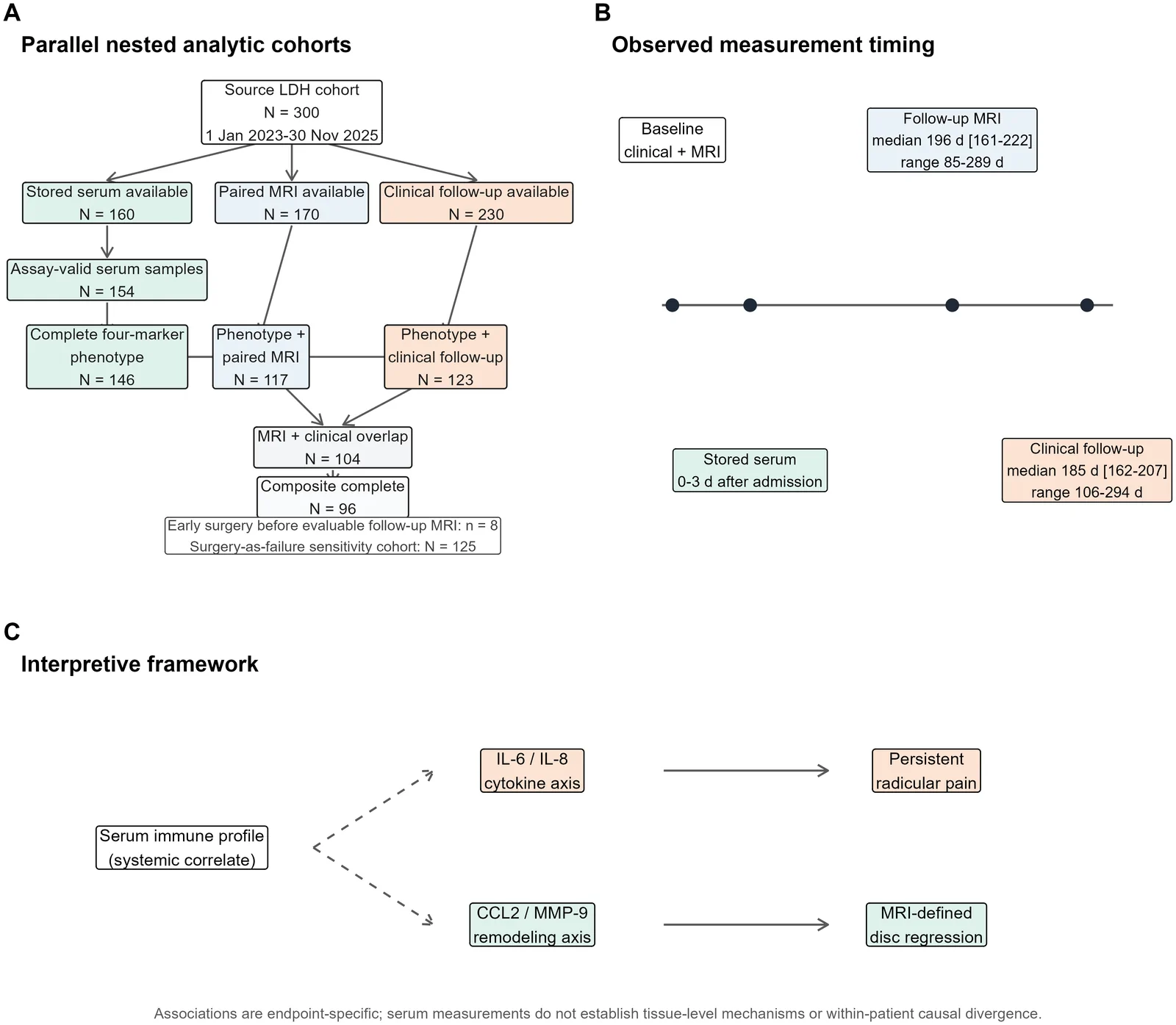
Parallel cohort architecture, observed measurement timing, and interpretive framework. **(A)** The source cohort branches separately into stored-serum, paired-MRI, and clinical-follow-up availability; endpoint-specific analyses then intersect phenotype assignment with each endpoint. Early surgery before evaluable MRI (n=8) is shown separately because it was treated as structural failure in sensitivity analysis. **(B)** Baseline-to-follow-up MRI timing was recalculated from MRI dates; the primary MRI cohort had a median interval of 196 [161-222] days (range 85-289). **(C)** Serum profiles are shown as systemic correlates of endpoint-specific outcomes; tissue-level mechanisms and within-patient causal divergence are not established.

The source cohort included 300 patients. Clinical follow-up was available in 230, paired MRI in 170, and stored serum in 160; 154 serum samples met assay-validity criteria and 146 had complete positive values for all four primary markers. Endpoint-specific cohorts comprised 117 patients for MRI regression and 123 for persistent pain, with 104 patients in the MRI-clinical overlap and 96 with complete exploratory composite data (**Figure 1**; **Supplementary Table 1**). Phenotype proportions were similar across the 146-patient phenotype cohort, MRI cohort, pain cohort, and overlap cohort (**Supplementary Table 1**). Multistage baseline comparisons identified differences at several selection steps, including symptom duration and morphology for serum availability and baseline VAS/morphology for clinical follow-up (**Supplementary Table 4**). Stabilized weighting reduced all reported absolute post-weighting standardized mean differences below 0.10; extreme raw MRI-stage weights required 1st-99th percentile truncation (**Supplementary Table 5**). Analytic availability is summarized graphically in **Supplementary Figure 1**, and weighting balance is shown in **Supplementary Figure 3**.

Baseline characteristics of the four-marker phenotype cohort are shown in **Table 1**. Median age was 44 years and 84 patients (57.5%) were male. Positive straight-leg raising and motor weakness differed across phenotypes; motor weakness was most frequent in the chemokine/MMP-dominant group. Several additional variables showed nontrivial effect-size imbalances despite nonsignificant omnibus P values, including baseline leg-pain VAS, CRP, and NLR. These patterns were treated as evidence of residual baseline heterogeneity rather than as proof of equivalence across classes.

**Table 1:** Baseline characteristics of the four-marker serum immune-phenotype cohort.

Characteristic	Overall	Low immune activation	Cytokine-dominant	Chemokine/MMP-dominant	P value	Effect size
Age, years	44 [37, 52]	40 [35, 51]	44 [37, 53]	44 [38, 50]	0.305	0.29
Sex					0.624	0.08
Female	62 (42.5%)	21 (45.7%)	15 (46.9%)	26 (38.2%)		
Male	84 (57.5%)	25 (54.3%)	17 (53.1%)	42 (61.8%)		
BMI, kg/m2	24.9 [22.0, 26.6]	25.4 [23.3, 27.0]	24.1 [21.7, 26.4]	24.6 [21.9, 25.9]	0.217	0.33
Smoking status					0.421	0.12
Never	98 (67.1%)	34 (73.9%)	23 (71.9%)	41 (60.3%)		
Former	24 (16.4%)	7 (15.2%)	3 (9.4%)	14 (20.6%)		
Current	24 (16.4%)	5 (10.9%)	6 (18.8%)	13 (19.1%)		
Diabetes, n (%)	9 (6.2%)	2 (4.3%)	3 (9.4%)	4 (5.9%)	0.678	0.08
Hypertension, n (%)	21 (14.4%)	9 (19.6%)	3 (9.4%)	9 (13.2%)	0.481	0.11
Symptom duration, days	39 [20, 80]	36 [16, 75]	44 [21, 83]	38 [21, 74]	0.693	0.14
Acute onset, n (%)	83 (56.8%)	26 (56.5%)	16 (50.0%)	41 (60.3%)	0.624	0.08
Baseline VAS leg pain	6.8 [5.6, 7.8]	6.5 [5.1, 7.1]	7.3 [6.2, 7.9]	6.8 [5.7, 8.0]	0.089	0.48
Baseline VAS back pain	3.8 [2.8, 4.8]	3.8 [2.9, 5.0]	4.0 [2.6, 5.2]	3.8 [2.8, 4.6]	0.870	0.14
Baseline ODI	67.1 [58.9, 73.8]	65.5 [56.7, 71.8]	66.7 [61.4, 74.3]	68.1 [60.0, 74.1]	0.610	0.16
Positive straight-leg raising, n (%)	94 (64.4%)	23 (50.0%)	26 (81.2%)	45 (66.2%)	0.016	0.24
Sensory deficit, n (%)	50 (34.2%)	13 (28.3%)	14 (43.8%)	23 (33.8%)	0.364	0.12
Motor weakness, n (%)	32 (21.9%)	3 (6.5%)	6 (18.8%)	23 (33.8%)	0.002	0.29
Target level					0.349	0.15
L3/4	16 (11.0%)	5 (10.9%)	6 (18.8%)	5 (7.4%)		
L4/5	60 (41.1%)	17 (37.0%)	16 (50.0%)	27 (39.7%)		
L5/S1	65 (44.5%)	23 (50.0%)	9 (28.1%)	33 (48.5%)		
Multilevel	5 (3.4%)	1 (2.2%)	1 (3.1%)	3 (4.4%)		
Herniation morphology					0.809	0.07
Protrusion	50 (34.2%)	18 (39.1%)	11 (34.4%)	21 (30.9%)		
Extrusion	71 (48.6%)	21 (45.7%)	14 (43.8%)	36 (52.9%)		
Sequestration	25 (17.1%)	7 (15.2%)	7 (21.9%)	11 (16.2%)		
Baseline herniation volume, cm3	0.38 [0.21, 0.61]	0.38 [0.21, 0.58]	0.40 [0.20, 0.69]	0.36 [0.21, 0.59]	0.913	0.19
Nerve root compression					0.955	0.08
None	15 (10.3%)	6 (13.0%)	2 (6.2%)	7 (10.3%)		
Contact	53 (36.3%)	16 (34.8%)	11 (34.4%)	26 (38.2%)		
Displacement	38 (26.0%)	13 (28.3%)	9 (28.1%)	16 (23.5%)		
Compression	40 (27.4%)	11 (23.9%)	10 (31.2%)	19 (27.9%)		
Pfirrmann grade	3 [3, 4]	3 [3, 4]	4 [3, 4]	3 [3, 4]	0.230	0.37
Modic type					0.893	0.09
None	94 (64.4%)	29 (63.0%)	19 (59.4%)	46 (67.6%)		
Type 1	23 (15.8%)	6 (13.0%)	6 (18.8%)	11 (16.2%)		
Type 2	27 (18.5%)	10 (21.7%)	7 (21.9%)	10 (14.7%)		
Type 3	2 (1.4%)	1 (2.2%)	0 (0.0%)	1 (1.5%)		
CRP, mg/L	2.6 [1.5, 4.0]	2.8 [1.7, 4.0]	2.9 [1.6, 4.0]	2.3 [1.5, 3.9]	0.669	0.38
NLR	2.00 [1.53, 2.58]	2.03 [1.51, 2.51]	2.16 [1.44, 2.89]	1.91 [1.56, 2.37]	0.613	0.34
NSAIDs before blood draw, n (%)	78 (53.4%)	21 (45.7%)	19 (59.4%)	38 (55.9%)	0.420	0.11
Oral steroid before blood draw, n (%)	3 (2.1%)	0 (0.0%)	1 (3.1%)	2 (2.9%)	0.597	0.10
Epidural steroid before blood draw, n (%)	7 (4.8%)	1 (2.2%)	1 (3.1%)	5 (7.4%)	0.535	0.11

Values are median [IQR] or n (%). P values are descriptive Kruskal-Wallis, Fisher exact, chi-square, or Monte Carlo chi-square tests as appropriate; no multiplicity adjustment was applied. Effect size is maximum pairwise standardized mean difference for continuous variables or Cramer’s V for categorical variables. Baseline P values were not used as the sole basis for covariate selection.

### 3.2 Outcome-blinded serum immune phenotyping

**Figure 2:**
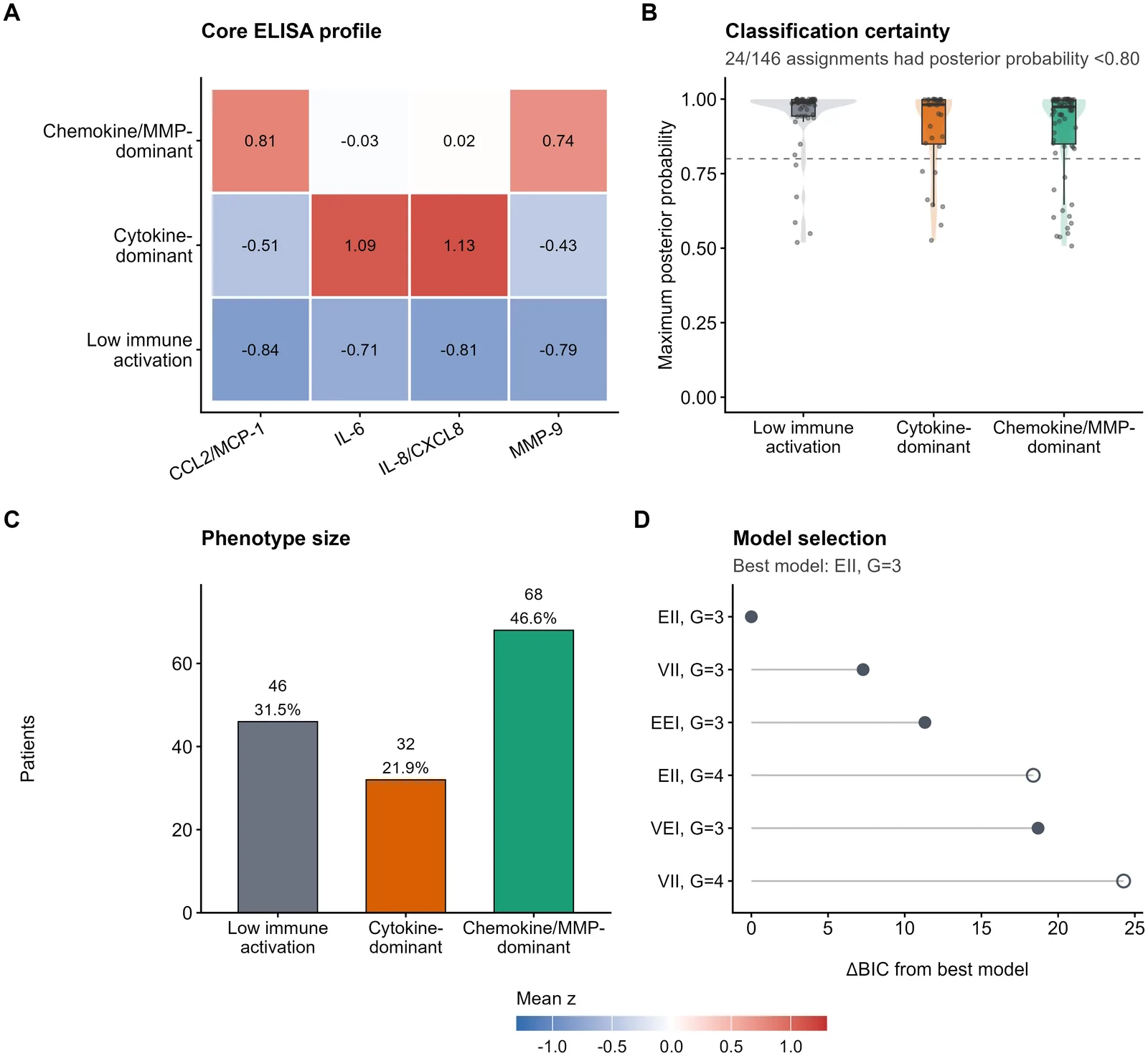
Data-driven serum immune phenotypes and model selection. **(A)** Mean standardized log-transformed IL-6, IL-8/CXCL8, CCL2/MCP-1, and MMP-9 profiles. **(B)** Maximum posterior assignment probabilities; the dashed line marks 0.80. **(C)** Class sizes. **(D)** BIC differences among leading candidate mclust solutions; EII with three classes had the best BIC (-1602.831), 7.285 points better than the next-best model. Clustering used no outcome variables.

Gaussian mixture modeling of standardized log-transformed IL-6, IL-8/CXCL8, CCL2/MCP-1, and MMP-9 identified three serum immune phenotypes (**Figure 2**). Low immune activation (n=46) showed lower standardized values across all four markers. The cytokine-dominant phenotype (n=32) showed higher IL-6 and IL-8/CXCL8 with relatively lower CCL2/MCP-1 and MMP-9. The chemokine/MMP-dominant phenotype (n=68) showed higher CCL2/MCP-1 and MMP-9 with near-average IL-6 and IL-8/CXCL8. Marker distributions by phenotype are shown in **Supplementary Figure 2**.

The EII three-class model was the BIC-preferred solution (BIC -1602.831), with a 7.285-point BIC advantage over the next-best VII three-class model (**Supplementary Table 3**). Median maximum posterior probability was 0.981; 24/146 assignments had posterior probability <0.80. Median subsample adjusted Rand index was 0.939 across 100 repetitions. Excluding lower-certainty assignments widened outcome uncertainty but did not reverse the main direction. Storage-adjusted reclustering agreed with the primary classification in 93.8% of patients (ARI 0.822), and adding MMP-3 as a fifth marker yielded 93.0% agreement (ARI 0.805) (**Supplementary Tables 3**, **S11**).

### 3.3 Immune phenotypes and MRI-defined disc regression

**Figure 3:**
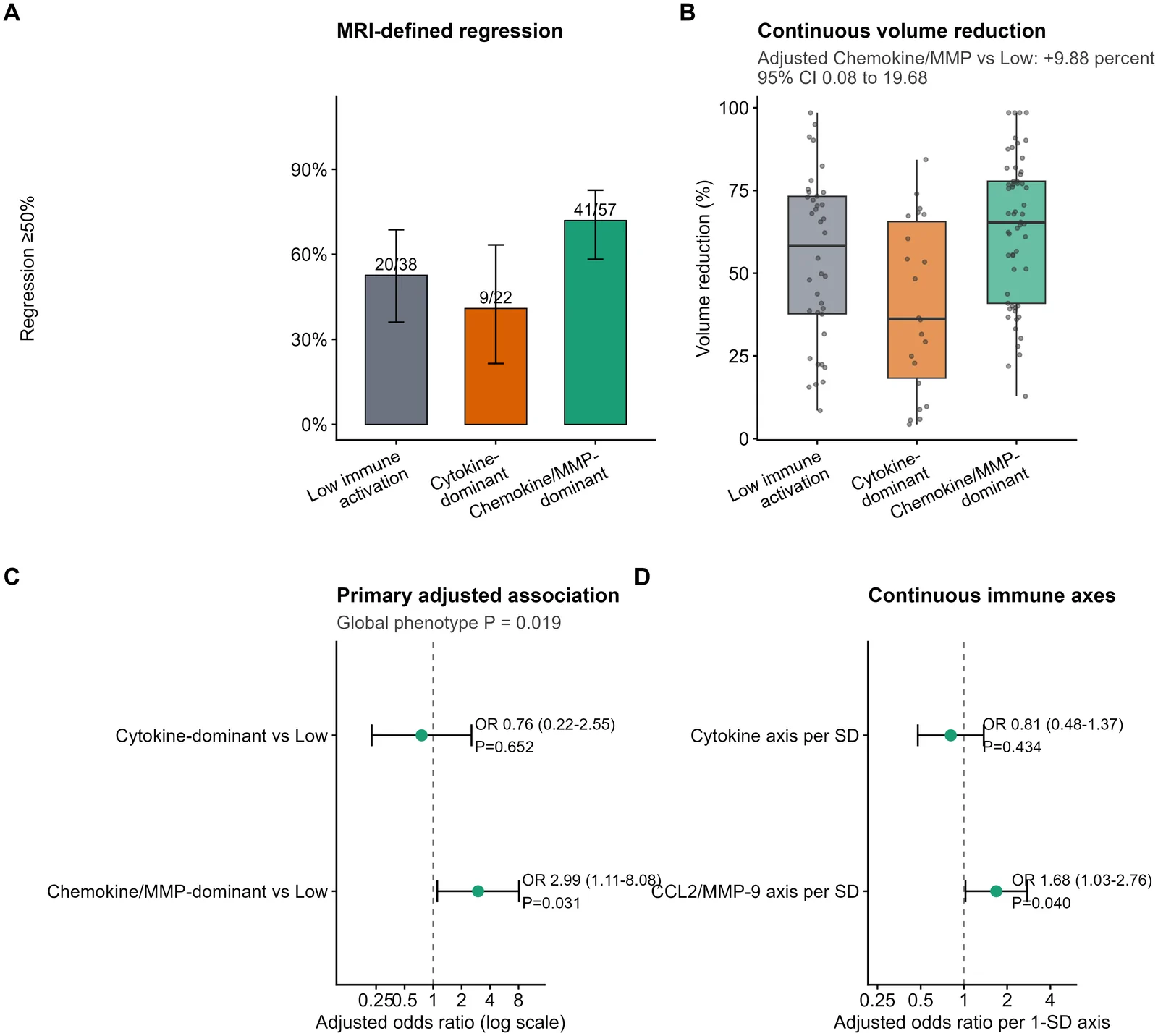
Serum immune phenotypes and MRI-defined disc regression in the phenotype plus paired-MRI cohort (N = 117). **(A)** Proportion with >=50% regression; error bars are 95% binomial confidence intervals. **(B)** Observed continuous percentage volume reduction; boxplots show median and interquartile range with individual observations. **(C)** Primary adjusted logistic odds ratios with 95% confidence intervals. **(D)** Adjusted continuous immune-axis odds ratios per 1-SD increase. The adjusted continuous volume model estimated 9.88 percentage points greater reduction for chemokine/MMP-dominant versus low immune activation (95% CI 0.08-19.68).

In the phenotype plus paired-MRI cohort (N = 117), the corrected baseline-to-follow-up MRI interval was 196 [161, 222] days (range 85-289). Median herniation-volume reduction was 58.4% in low immune activation, 36.2% in the cytokine-dominant phenotype, and 65.4% in the chemokine/MMP-dominant phenotype (P = 0.002; **Table 2**; **Figure 3**). MRI regression >=50% occurred in 70 patients (59.8%) and was most frequent in the chemokine/MMP-dominant group (52.6%, 40.9%, and 71.9%, respectively; P = 0.023). The secondary >=70% threshold showed the same ordering (P = 0.020).

**Table 2:** Endpoint-specific MRI and clinical outcomes across serum immune phenotypes.

Outcome	Overall	Low immune activation	Cytokine-dominant	Chemokine/MMP-dominant	P value
Phenotype + paired MRI cohort, N = 117					
MRI follow-up interval, days	196 [161, 222]	199 [162, 226]	192 [158, 208]	198 [160, 220]	0.689
Herniation volume reduction, %	61.9 [36.7, 75.6]	58.4 [37.7, 73.2]	36.2 [18.3, 65.6]	65.4 [40.9, 77.8]	0.002
MRI regression >=50%, n (%)	70 (59.8%)	20 (52.6%)	9 (40.9%)	41 (71.9%)	0.023
MRI regression >=70%, n (%)	40 (34.2%)	14 (36.8%)	2 (9.1%)	24 (42.1%)	0.020
Phenotype + clinical follow-up cohort, N = 123					
Clinical follow-up interval, days	185 [162, 207]	191 [158, 207]	180 [160, 203]	189 [168, 211]	0.582
6-month VAS leg pain	2.5 [1.2, 4.0]	2.0 [1.0, 3.6]	3.6 [2.7, 4.9]	2.2 [1.3, 3.7]	0.018
VAS leg pain improvement	4.2 [3.0, 5.5]	4.1 [2.8, 5.1]	3.5 [2.8, 4.4]	4.9 [3.8, 5.9]	0.003
6-month ODI	30.7 [20.4, 41.1]	26.9 [20.4, 36.6]	41.4 [30.7, 45.7]	29.8 [19.1, 35.9]	0.017
ODI improvement	35.6 [26.4, 44.1]	35.9 [30.5, 43.5]	31.2 [20.7, 36.7]	36.2 [28.5, 51.0]	0.055
Persistent radicular pain, n (%)	52 (42.3%)	15 (38.5%)	17 (63.0%)	20 (35.1%)	0.046
Exploratory overlap cohort with complete composite data, N = 96					
Exploratory composite favorable recovery, n (%)	42 (43.8%)	14 (48.3%)	4 (20.0%)	24 (51.1%)	0.054

MRI outcomes use the phenotype plus paired-MRI cohort (N = 117), clinical outcomes use the phenotype plus clinical follow-up cohort (N = 123), and the exploratory composite uses the overlap cohort with complete component data (N = 96). MRI follow-up interval is recalculated from baseline and follow-up MRI dates. Adjusted continuous outcome models are presented in **Supplementary Table 9**.

In the primary adjusted logistic model, the chemokine/MMP-dominant phenotype was associated with MRI regression relative to low immune activation (OR 2.99, 95% CI 1.11-8.08; P = 0.031; global phenotype P = 0.019; **Table 3**). The adjusted continuous structural model estimated 9.88 percentage points greater volume reduction for chemokine/MMP-dominant versus low immune activation (95% CI 0.08-19.68; P = 0.048; global phenotype P = 0.002). The continuous CCL2/MMP-9 remodeling axis was also associated with regression (OR per 1-SD 1.68, 95% CI 1.03-2.76; P = 0.040). Sensitivity analyses supported the overall direction but showed meaningful imprecision: the chemokine/MMP contrast was OR 2.60 (95% CI 0.95-7.11; P = 0.062) with nonlinear age adjustment, OR 2.80 (0.96-8.11; P = 0.058) after excluding posterior probabilities <0.80, and OR 2.23 (0.88-5.62; P = 0.089) when eight early surgeries were treated as structural failures. With additional adjustment for initial treatment plan in the surgery-as-failure cohort, the OR was 2.79 (1.04-7.49; P = 0.042). Firth, modified-Poisson, IPW, storage-adjusted, and five-marker analyses are reported in **Supplementary Table 8** and **Supplementary Figure 4**.

**Table 3:** Primary associations of serum immune phenotypes with MRI regression, persistent pain, and exploratory composite recovery.

Endpoint	Model	Effect	N	Events	Odds ratio (95% CI)	Pairwise P	Global phenotype P
MRI regression >=50%	Unadjusted	Cytokine-dominant vs Low	117	70	0.62 (0.22 to 1.80)	0.383	0.022
MRI regression >=50%	Unadjusted	Chemokine/MMP-dominant vs Low	117	70	2.31 (0.98 to 5.45)	0.057	0.022
MRI regression >=50%	Model 2: primary adjusted	Cytokine-dominant vs Low	117	70	0.76 (0.22 to 2.55)	0.652	0.019
MRI regression >=50%	Model 2: primary adjusted	Chemokine/MMP-dominant vs Low	117	70	2.99 (1.11 to 8.08)	0.031	0.019
Persistent radicular pain	Unadjusted	Cytokine-dominant vs Low	123	52	2.72 (0.99 to 7.49)	0.053	0.047
Persistent radicular pain	Unadjusted	Chemokine/MMP-dominant vs Low	123	52	0.86 (0.37 to 2.01)	0.736	0.047
Persistent radicular pain	Primary adjusted	Cytokine-dominant vs Low	123	52	2.23 (0.65 to 7.65)	0.203	0.003
Persistent radicular pain	Primary adjusted	Chemokine/MMP-dominant vs Low	123	52	0.30 (0.09 to 0.95)	0.042	0.003
Exploratory composite favorable recovery	Unadjusted	Cytokine-dominant vs Low	96	42	0.27 (0.07 to 1.00)	0.050	0.044
Exploratory composite favorable recovery	Unadjusted	Chemokine/MMP-dominant vs Low	96	42	1.12 (0.44 to 2.82)	0.813	0.044
Exploratory composite favorable recovery	Primary adjusted	Cytokine-dominant vs Low	96	42	0.44 (0.10 to 1.99)	0.285	0.062
Exploratory composite favorable recovery	Primary adjusted	Chemokine/MMP-dominant vs Low	96	42	2.07 (0.63 to 6.84)	0.231	0.062

Model 1 is unadjusted. MRI Model 2 adjusts for age, sex, body mass index, morphology, baseline herniation volume, and corrected baseline-to-follow-up MRI interval. Persistent-pain Model 2 adjusts for age, sex, body mass index, baseline leg-pain VAS, motor weakness as a marker of neurological severity, and morphology. The composite is exploratory. Wald 95% confidence intervals and pairwise P values are shown for standard logistic models; global phenotype P values are likelihood-ratio tests. Full sensitivity analyses, selection weighting, continuous outcomes, missingness, and model diagnostics are reported in **Supplementary Tables 4-S12**. No multiplicity adjustment was applied.

### 3.4 Immune phenotypes and persistent radicular pain

**Figure 4:**
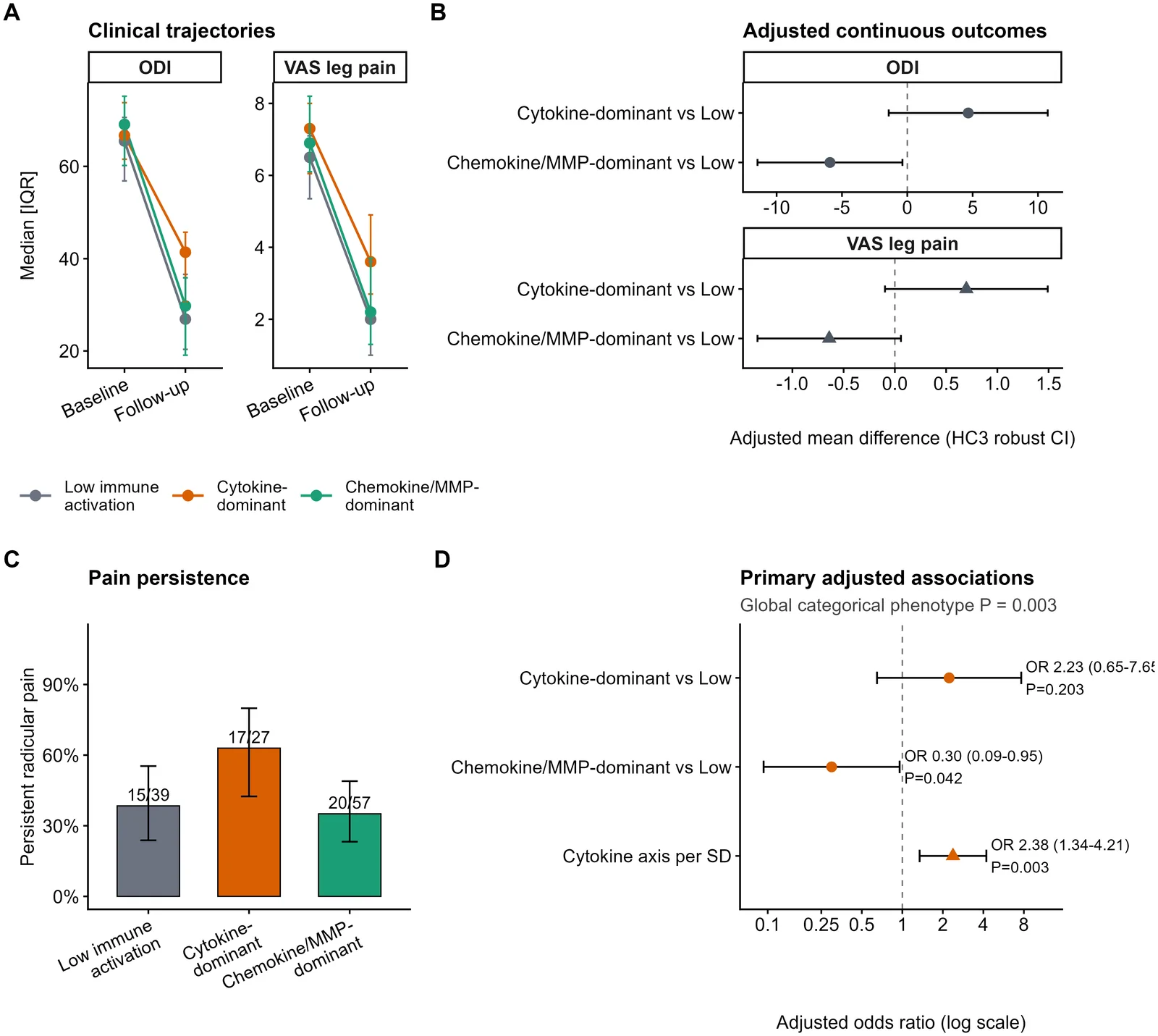
Serum immune phenotypes and persistent radicular pain in the phenotype plus clinical follow-up cohort (N = 123). **(A)** Median VAS leg-pain and ODI trajectories; error bars are interquartile ranges. **(B)** Baseline-adjusted continuous 6-month VAS and ODI differences with HC3 robust 95% confidence intervals. **(C)** Persistent-pain proportions with 95% binomial confidence intervals. **(D)** Primary adjusted categorical phenotype odds ratios and the continuous cytokine-axis odds ratio; all error bars are 95% confidence intervals.

In the phenotype plus clinical follow-up cohort (N = 123), the median recorded follow-up interval was 185 [162, 207] days. Unadjusted 6-month VAS leg pain and ODI were highest in the cytokine-dominant phenotype (**Table 2**; **Figure 4**). In adjusted continuous models, the cytokine-dominant versus low contrast for 6-month VAS was +0.70 points (95% CI -0.10 to 1.49; P = 0.085), whereas the chemokine/MMP-dominant contrast was -0.64 points (-1.34 to 0.06; P = 0.073). For 6-month ODI (N = 115), the corresponding adjusted differences were +4.66 (-1.42 to 10.75; P = 0.133) and -5.92 (-11.46 to -0.37; P = 0.036), respectively (**Supplementary Table 9**).

Persistent radicular pain occurred in 52/123 patients (42.3%) and was most frequent in the cytokine-dominant phenotype (38.5% low immune activation, 63.0% cytokine-dominant, 35.1% chemokine/MMP-dominant; P = 0.046; **Table 2**; **Figure 4**). In the primary adjusted model, the global phenotype likelihood-ratio P value was 0.0029. However, class-specific estimates were imprecise: cytokine-dominant versus low OR 2.23 (95% CI 0.65-7.65; P = 0.203) and chemokine/MMP-dominant versus low OR 0.30 (0.09-0.95; P = 0.042). The latter contrast was attenuated in several sensitivity analyses, including clinical-stage IPW (P = 0.069), combined source-to-clinical IPW (P = 0.106), five-marker clustering (P = 0.100), restriction to patients without surgery by 6 months (P = 0.055), and extreme-case missing-outcome analyses. These results support phenotype-level heterogeneity but not a precise standalone class effect. The corresponding sensitivity estimates are shown in **Supplementary Figure 5**.

The continuous cytokine axis provided the clearest pain association: each 1-SD increase was associated with higher odds of persistent pain (OR 2.38, 95% CI 1.34-4.21; P = 0.0029), whereas the CCL2/MMP-9 remodeling axis showed an imprecise inverse association (OR 0.62, 95% CI 0.37-1.07; P = 0.084; **Supplementary Table 10**). Thus, persistent pain was more consistently represented by a graded cytokine signal than by a single categorical phenotype.

### 3.5 Exploratory overlap analyses, attrition, and model diagnostics

The MRI-clinical overlap included 104 patients. Joint outcome states are reported descriptively in **Supplementary Table 1**. The exploratory composite favorable-recovery endpoint was complete in 96 patients and did not show a conventionally significant adjusted global phenotype association (P = 0.062; **Table 3**). The eight composite exclusions were attributable to missing follow-up ODI. Follow-up treatment trajectories, including epidural steroid injection, analgesic exposure, rehabilitation adherence, surgery, and rescue visits, are summarized in **Supplementary Table 7**. Endpoint-specific missingness and composite completeness are detailed in **Supplementary Table 6**.

No primary adjusted-model covariate was missing within either endpoint-specific cohort. MRI-model diagnostics showed low collinearity (maximum VIF 1.90) but evidence of age nonlinearity (quadratic test P = 0.030); the corresponding sensitivity estimate was therefore reported. The pain model had limited information (52 events; 9 predictor degrees of freedom), low collinearity (maximum VIF 1.44), and a Hosmer-Lemeshow P value of 0.027, indicating potential lack of fit; Firth and influence-exclusion analyses were used as stability checks (**Supplementary Table 12**). No internal prediction-performance analysis is presented because prediction was not a study objective.

## 4 Discussion

### 4.1 Principal findings

This study identified three outcome-blinded serum immune phenotypes in symptomatic LDH, but the results support endpoint-specific associations rather than definitive biological subtypes. The chemokine/MMP-dominant phenotype showed the clearest association with MRI-defined regression in the primary model and in adjusted continuous volume reduction. However, nonlinear-age, early-surgery, and classification-certainty analyses widened the class-specific confidence intervals. The categorical pain analysis also showed phenotype-level heterogeneity with imprecise pairwise contrasts, whereas the continuous IL-6/IL-8 cytokine axis was more clearly associated with persistent pain. Because the MRI and pain analyses used partially overlapping cohorts and the exploratory overlap composite was not statistically conclusive, these findings do not establish two divergent recovery pathways within the same patients.

### 4.2 Relationship to spontaneous resorption literature

The MRI regression finding is consistent with the broader literature that spontaneous disc regression is common but biologically selective. Chiu and colleagues found that regression probabilities differed markedly by herniation morphology, with higher regression in sequestration and extrusion than in protrusion or bulging (6). Zhong and colleagues similarly reported a substantial pooled incidence of spontaneous resorption in conservatively treated LDH (7). More recent reviews have emphasized that disc morphology, migration, vascular access, and inflammatory factors jointly shape the likelihood of resorption (10, 26). In the present data, the association was directionally preserved across several sensitivity analyses but was attenuated when age nonlinearity, early surgery, or classification certainty were handled more conservatively. This does not prove that the serum profile is upstream of regression, and residual confounding by baseline severity remains plausible.

A plausible biological interpretation is that the chemokine/MMP-dominant phenotype reflects a systemic correlate of monocyte/macrophage recruitment and matrix remodeling. CCL2/MCP-1 is a central chemotactic signal for monocytes through CCR2 biology, and chemokine gradients are fundamental to leukocyte trafficking into inflamed tissue (27, 28). Human intervertebral disc tissue can spontaneously produce MCP-1 and IL-8, and disc cells can generate MCP-1 shortly after experimental herniation (15, 16). Experimental work has shown that MMP-dependent signaling can generate macrophage chemoattractant activity in herniated disc resorption models, while human herniated tissue studies link macrophage infiltration with matrix metalloproteinase production (17, 18). The present serum findings sit within that biological frame, but they are systemic correlates rather than direct measurements of disc tissue, macrophage recruitment, CCR2 signaling, or local MMP activity.

The role of macrophages deserves particular attention. Reviews of disc resorption describe macrophage infiltration, phagocytosis, angiogenesis, and matrix degradation as recurring elements of spontaneous regression (8, 9). Contemporary immunology reviews have also framed the disc as an immune-modulated microenvironment rather than a purely degenerative tissue compartment (29). Macrophages may be injurious or reparative depending on context, activation state, and timing, a point emphasized in recent disc immunobiology literature (30). The chemokine/MMP-dominant serum profile therefore provides a biologically coherent association signal, but it should not be interpreted as direct evidence of macrophage-mediated tissue clearance.

### 4.3 Inflammation, pain, and the cytokine axis

The pain findings should be interpreted with more restraint. The cytokine-dominant phenotype had the highest unadjusted frequency of persistent radicular pain, but the adjusted pairwise comparison against low immune activation was not statistically robust. In contrast, the continuous cytokine axis based on IL-6 and IL-8/CXCL8 was associated with persistent pain. This distinction matters. It suggests that pain persistence may be better represented by a graded cytokine-inflammatory burden than by membership in one categorical phenotype. Prior prospective work showed that patients with chronic lumbar radicular pain after disc herniation had higher serum IL-6 and IL-8 than those with low pain at follow-up (19). A broader inflammatory serum protein profiling study also found that patients with severe pain one year after disc herniation had a distinct systemic inflammatory profile (20). Our findings are compatible with those observations. Importantly, categorical pairwise estimates were unstable across several sensitivity analyses, whereas the continuous cytokine axis was less dependent on class assignment.

The cytokine-pain association is biologically plausible, but the present data remain observational. IL-6 has been implicated in pathological pain through peripheral and central neuroimmune pathways (31). Neuroinflammation can sustain chronic pain by increasing neuronal excitability and central sensitization, particularly when cytokine and chemokine signaling persists beyond the acute injury phase (32). IL-8/CXCL8 is both a chemokine and a proinflammatory mediator, and its elevation in radicular pain studies suggests that it may mark a clinically relevant inflammatory state rather than nonspecific systemic noise (19). In the present data, the cytokine axis was associated with persistent pain even when categorical phenotype contrasts were imprecise. This supports a graded cytokine-linked pain signal without establishing a discrete clinical risk class.

### 4.4 Structural recovery and pain recovery are not equivalent

The divergence between MRI regression and pain persistence is clinically important. LDH symptoms are influenced by mechanical compression, chemical radiculitis, nerve-root injury, central sensitization, baseline neurological deficit, treatment exposure, and psychosocial context (1, 4). Surgical and nonoperative outcome studies also show that clinical recovery trajectories are heterogeneous and not completely explained by imaging features alone (2, 3). Our endpoint-specific results fit this complexity, but the MRI and pain cohorts only partially overlapped. The data therefore support different association patterns across endpoints rather than proving that structural clearance and pain persistence diverge mechanistically within individual patients.

This distinction may also explain why anti-inflammatory treatment in LDH remains conceptually difficult. Suppressing inflammation may reduce pain in some patients, but the inflammatory response also participates in removal of herniated tissue. A recent Frontiers in Immunology article framed this tension as a need to reconcile pain control with preservation of reparative resorption biology (33). Our data do not estimate treatment effects and should not guide anti-inflammatory therapy. The observed serum patterns are better viewed as hypothesis-generating correlates of structural and pain outcomes than as evidence for a treatment-preservation strategy.

### 4.5 Clinical and translational implications

The most immediate implication is not clinical deployment, but refinement of the biological phenotype used in LDH research. Conventional predictors of resorption, such as extrusion, sequestration, migration, and rim enhancement, remain important (10, 12, 26). A serum chemokine/MMP profile may eventually complement imaging phenotypes in mechanistic research, but the present single-center data do not justify patient-level stratification, treatment selection, or targeted imaging schedules. Clinical implementation would require standardized biomarker procedures, clearly specified clinical use cases, and independent multicenter validation.

A second implication is methodological. Serum biomarker studies in LDH often focus on individual markers, which can be difficult to interpret because IL-6, IL-8, CCL2, and MMPs are pleiotropic and context-dependent (13, 21). Outcome-blinded clustering and continuous-axis analyses provided complementary representations of the same baseline marker panel. Their partial convergence supports further study, but neither approach eliminates uncertainty introduced by data-driven class definition, selection, multiple analyses, or limited sample size.

### 4.6 Strengths

The study has several strengths. It integrated baseline serum immune measurements with paired-MRI structural outcomes and longitudinal pain/function outcomes, and phenotype derivation excluded all outcomes. The analysis directly evaluated multiple selection processes, informative early surgery, classification certainty, storage duration, alternative five-marker clustering, common-outcome risk ratios, adjusted continuous outcomes, and influential observations. Reporting endpoint-specific cohorts avoided discarding available data solely to force all outcomes into the smallest overlap set. These strengths improve transparency, but they do not remove the observational and measurement limitations described below.

### 4.7 Limitations

Several limitations materially constrain interpretation. First, this was a single-center observational study without prospective registration or an outcome analysis plan registered before outcome ascertainment; no multiplicity adjustment was applied, and secondary/sensitivity P values are exploratory. Second, the endpoint-specific cohorts were generated through non-random serum, MRI, and clinical follow-up availability. Weighting improved measured covariate balance, but residual selection and collider bias remain possible, particularly because early surgery was phenotype-imbalanced. Third, serum markers cannot localize immune activity to herniated disc tissue or the affected nerve root, and no paired tissue, single-cell, or local-fluid validation was available. Fourth, storage duration correlated modestly with CCL2 and MMP-9; storage-adjusted clustering was reassuring, and the residual clinical serum used for ELISA had not undergone repeated freeze-thaw cycling before assay. Fifth, an archived inter-reader reliability summary supported agreement across categorical and volumetric MRI assessments, but it did not retain the double-read sample size, exact ICC parameterization, intra-reader repeat data, or harmonized scanner/sequence metadata; recorded volume estimates also arose from more than one measurement method. Sixth, the multivariable information base was modest, age nonlinearity affected the MRI estimate, and the pain model showed potential lack of fit. Finally, the categorical pain contrasts were sensitive to analytic choices, and the partially overlapping endpoint cohorts do not establish within-patient divergence of structural and pain pathways.

### 4.8 Future directions

Future studies should use prospective multicenter cohorts with prospectively standardized serum processing, harmonized MRI acquisition and volumetry, and follow-up windows defined in advance within the study protocol. Repeated serum sampling and paired surgical tissue could test whether circulating chemokine/MMP profiles correspond to local macrophage/CCR2 signaling, matrix metalloproteinase activity, or other tissue states. Independent replication should evaluate both categorical phenotype stability and continuous immune axes, while prediction research should be treated as a separate objective with full internal and external validation.

## 5 Conclusion

In this single-center observational cohort, a chemokine/MMP-dominant baseline serum phenotype was associated with MRI-defined disc regression during nonoperative follow-up, although several sensitivity analyses widened uncertainty around the class-specific estimate. Persistent radicular pain was more consistently associated with a continuous IL-6/IL-8 cytokine axis than with categorical phenotype membership. These findings identify hypothesis-generating systemic immune correlates of structural and pain outcomes rather than causal tissue mechanisms or a clinical decision tool.

## Data Availability

The original contributions presented in the study are included in the article/**Supplementary Material**. Further inquiries can be directed to the corresponding author.
