# Supplementary material for: Data-driven serum cytokine and chemokine phenotypes are associated with MRI-defined disc regression and persistent radicular pain in lumbar disc herniation
Source: Front Immunol. 2026 Sep 3;17:1928963. doi: 10.3389/fimmu.2026.1928963 (PMC13581728; doi:10.3389/fimmu.2026.1928963)
Supplement: Supplementary file 1 [file Table1.docx]

**Supplementary Materials**

Supplementary tables are presented before supplementary figures. All analyses use the endpoint-specific cohorts and statistical definitions described in the manuscript.

**Supplementary Tables**

**Supplementary Table S1. Cohort construction and phenotype distribution across endpoint-specific analytic sets.**

| **Cohort/analytic set** | **N** |
| --- | --- |
| Full clinical LDH cohort | 300 |
| Clinical follow-up available | 230 |
| Paired MRI available | 170 |
| ELISA available | 160 |
| Assay-valid serum samples | 154 |
| Complete four-marker phenotype cohort | 146 |
| Phenotype + paired MRI primary cohort | 117 |
| Phenotype + clinical follow-up primary cohort | 123 |
| MRI + clinical overlap cohort | 104 |
| Complete exploratory composite | 96 |
| Early surgery before evaluable follow-up MRI within phenotype cohort | 8 |
| Surgery-as-failure structural sensitivity cohort | 125 |

*Phenotype distribution within the principal nested cohorts:*

| **Cohort** | **N** | **Low** | **Cytokine-dominant** | **Chemokine/MMP-dominant** |
| --- | --- | --- | --- | --- |
| Four-marker phenotype cohort | 146 | 46 (31.5%) | 32 (21.9%) | 68 (46.6%) |
| MRI primary cohort | 117 | 38 (32.5%) | 22 (18.8%) | 57 (48.7%) |
| Persistent-pain primary cohort | 123 | 39 (31.7%) | 27 (22.0%) | 57 (46.3%) |
| MRI + clinical overlap cohort | 104 | 33 (31.7%) | 21 (20.2%) | 50 (48.1%) |

*Joint MRI-regression/persistent-pain states in the MRI-clinical overlap cohort (N=104):*

| **immune_phenotype** | **Joint_state** | **n** | **denom** | **percent** |
| --- | --- | --- | --- | --- |
| Low immune activation | MRI regression + no persistent pain | 15 | 33 | 45.455 |
| Low immune activation | MRI regression + persistent pain | 4 | 33 | 12.121 |
| Low immune activation | No MRI regression + no persistent pain | 7 | 33 | 21.212 |
| Low immune activation | No MRI regression + persistent pain | 7 | 33 | 21.212 |
| Cytokine-dominant | MRI regression + no persistent pain | 4 | 21 | 19.048 |
| Cytokine-dominant | MRI regression + persistent pain | 4 | 21 | 19.048 |
| Cytokine-dominant | No MRI regression + no persistent pain | 4 | 21 | 19.048 |
| Cytokine-dominant | No MRI regression + persistent pain | 9 | 21 | 42.857 |
| Chemokine/MMP-dominant | MRI regression + no persistent pain | 26 | 50 | 52.000 |
| Chemokine/MMP-dominant | MRI regression + persistent pain | 11 | 50 | 22.000 |
| Chemokine/MMP-dominant | No MRI regression + no persistent pain | 7 | 50 | 14.000 |
| Chemokine/MMP-dominant | No MRI regression + persistent pain | 6 | 50 | 12.000 |

**Supplementary Table S2. ELISA assay-validity, timing, and storage characteristics.**

| **Item** | **Value** |
| --- | --- |
| Stored serum available | 160 |
| Collection timing after admission, median [IQR], days | 2 [1, 2] |
| Collection timing range, days | 0-3 |
| Assay-valid serum samples | 154/160 |
| Complete four-marker phenotype inputs | 146 |
| ELISA plate count | 8 |
| Sample storage duration, median [IQR], months | 22 [13, 32] |
| Duplicate CV maximum, median [IQR], % | 6.8 [5.4, 8.3] |
| Repeated freeze-thaw cycles before ELISA | None |
| Serum source | Residual serum remaining after routine clinical blood collection |

*Storage duration by phenotype:*

| **Phenotype** | **N** | **Median** | **Q1** | **Q3** | **Min** | **Max** | **P value** |
| --- | --- | --- | --- | --- | --- | --- | --- |
| Low immune activation | 46 | 26 | 18.250 | 33.750 | 7 | 46 | 0.063 |
| Cytokine-dominant | 32 | 25 | 12.000 | 31.250 | 8 | 44 | 0.063 |
| Chemokine/MMP-dominant | 68 | 19 | 13.000 | 29.250 | 5 | 42 | 0.063 |

*Spearman correlations between storage duration and marker concentrations:*

| **Marker** | **N** | **Spearman rho** | **P value** |
| --- | --- | --- | --- |
| il6_pg_ml | 146 | 0.082 | 0.326 |
| il8_pg_ml | 146 | -0.122 | 0.143 |
| ccl2_pg_ml | 146 | -0.165 | 0.047 |
| mmp9_ng_ml | 146 | -0.230 | 0.005 |
| mmp3_ng_ml | 142 | -0.081 | 0.337 |

*The ELISA specimens were residual serum remaining after routine clinical blood collection, and no repeated freeze-thaw cycles occurred before ELISA testing.*

**Supplementary Table S3. Gaussian mixture model selection, classification certainty, and stability.**

| **Metric** | **Value** |
| --- | --- |
| Primary model | EII |
| Primary class number | 3 |
| Primary EII G=3 BIC | -1602.831 |
| Entropy | 0.793 |
| Median maximum posterior probability | 0.981 |
| Posterior <0.70, n | 20 |
| Posterior <0.80, n | 24 |
| Posterior <0.90, n | 38 |
| Subsample ARI median | 0.939 |
| Subsample ARI IQR | 0.078 |
| Subsample repetitions | 100 |

*Leading candidate models:*

| **G** | **Model** | **BIC** | **Delta BIC** | **Minimum class n** | **Minimum class %** | **Admissible** |
| --- | --- | --- | --- | --- | --- | --- |
| 3 | EII | -1602.831 | 0.000 | 32 | 21.9% | True |
| 3 | VII | -1610.116 | 7.285 | 29 | 19.9% | True |
| 3 | EEI | -1614.147 | 11.316 | 36 | 24.7% | True |
| 4 | EII | -1621.212 | 18.381 | 6 | 4.1% | False |
| 3 | VEI | -1621.523 | 18.692 | 28 | 19.2% | True |
| 4 | VII | -1627.101 | 24.270 | 6 | 4.1% | False |
| 2 | EVE | -1631.360 | 28.529 | 66 | 45.2% | True |
| 4 | EEI | -1632.973 | 30.142 | 8 | 5.5% | False |
| 3 | EVI | -1633.779 | 30.948 | 40 | 27.4% | True |
| 2 | VVE | -1636.304 | 33.473 | 63 | 43.2% | True |

**Supplementary Table S4. Multistage baseline comparisons for analytic-set availability.**

| **Selection stage** | **Variable** | **Level** | **Observed** | **Not observed** | **P value** |
| --- | --- | --- | --- | --- | --- |
| Serum availability in source cohort | Age, years |  | 43.5 [37.0, 51.2] | 46.0 [38.0, 55.2] | 0.082 |
| Serum availability in source cohort | Sex | Female | 65 (40.6%) | 50 (35.7%) | 0.451 |
| Serum availability in source cohort | Sex | Male | 95 (59.4%) | 90 (64.3%) | 0.451 |
| Serum availability in source cohort | BMI, kg/m2 |  | 24.6 [22.0, 26.4] | 24.9 [22.8, 27.4] | 0.156 |
| Serum availability in source cohort | Symptom duration, days |  | 44.0 [20.0, 84.2] | 64.0 [26.8, 109.5] | 0.019 |
| Serum availability in source cohort | Baseline leg-pain VAS |  | 6.8 [5.6, 7.8] | 6.5 [5.6, 7.4] | 0.466 |
| Serum availability in source cohort | Baseline ODI |  | 66.9 [59.0, 73.5] | 66.8 [59.6, 72.3] | 0.615 |
| Serum availability in source cohort | Herniation morphology | Extrusion | 75 (46.9%) | 59 (42.1%) | 0.011 |
| Serum availability in source cohort | Herniation morphology | Protrusion | 56 (35.0%) | 69 (49.3%) | 0.011 |
| Serum availability in source cohort | Herniation morphology | Sequestration | 29 (18.1%) | 12 (8.6%) | 0.011 |
| Serum availability in source cohort | Baseline herniation volume, cm3 |  | 0.4 [0.2, 0.6] | 0.3 [0.2, 0.5] | 0.077 |
| Serum availability in source cohort | Initial treatment plan | Conservative | 103 (64.4%) | 86 (61.4%) | 0.842 |
| Serum availability in source cohort | Initial treatment plan | Surgery planning | 16 (10.0%) | 14 (10.0%) | 0.842 |
| Serum availability in source cohort | Initial treatment plan | Nerve root block/injection | 41 (25.6%) | 40 (28.6%) | 0.842 |
| Serum availability in source cohort | Distance to hospital, km |  | 14.1 [7.7, 31.3] | 13.7 [8.0, 25.5] | 0.582 |
| Serum availability in source cohort | Insurance type | Urban-rural resident insurance | 51 (31.9%) | 45 (32.1%) | 0.720 |
| Serum availability in source cohort | Insurance type | Urban employee insurance | 77 (48.1%) | 74 (52.9%) | 0.720 |
| Serum availability in source cohort | Insurance type | Other | 5 (3.1%) | 5 (3.6%) | 0.720 |
| Serum availability in source cohort | Insurance type | Self-pay | 22 (13.8%) | 14 (10.0%) | 0.720 |
| Serum availability in source cohort | Insurance type | Commercial supplemental | 5 (3.1%) | 2 (1.4%) | 0.720 |
| Assay-valid serum among serum-available patients | Age, years |  | 43.0 [37.0, 52.0] | 46.0 [40.8, 49.0] | 0.836 |
| Assay-valid serum among serum-available patients | Sex | Female | 64 (41.6%) | 1 (16.7%) | 0.402 |
| Assay-valid serum among serum-available patients | Sex | Male | 90 (58.4%) | 5 (83.3%) | 0.402 |
| Assay-valid serum among serum-available patients | BMI, kg/m2 |  | 24.6 [21.9, 26.4] | 24.5 [23.5, 26.1] | 0.903 |
| Assay-valid serum among serum-available patients | Symptom duration, days |  | 44.0 [20.0, 84.0] | 45.0 [30.8, 75.8] | 0.689 |
| Assay-valid serum among serum-available patients | Baseline leg-pain VAS |  | 6.8 [5.6, 7.8] | 7.2 [7.0, 8.1] | 0.252 |
| Assay-valid serum among serum-available patients | Baseline ODI |  | 66.9 [58.8, 73.6] | 65.5 [63.6, 71.9] | 0.571 |
| Assay-valid serum among serum-available patients | Herniation morphology | Extrusion | 73 (47.4%) | 2 (33.3%) | 0.858 |
| Assay-valid serum among serum-available patients | Herniation morphology | Protrusion | 53 (34.4%) | 3 (50.0%) | 0.858 |
| Assay-valid serum among serum-available patients | Herniation morphology | Sequestration | 28 (18.2%) | 1 (16.7%) | 0.858 |
| Assay-valid serum among serum-available patients | Baseline herniation volume, cm3 |  | 0.4 [0.2, 0.6] | 0.2 [0.1, 0.6] | 0.350 |
| Assay-valid serum among serum-available patients | Initial treatment plan | Surgery planning | 16 (10.4%) | 0 (0.0%) | 1.000 |
| Assay-valid serum among serum-available patients | Initial treatment plan | Conservative | 99 (64.3%) | 4 (66.7%) | 1.000 |
| Assay-valid serum among serum-available patients | Initial treatment plan | Nerve root block/injection | 39 (25.3%) | 2 (33.3%) | 1.000 |
| Assay-valid serum among serum-available patients | Distance to hospital, km |  | 14.2 [7.8, 32.0] | 11.5 [7.4, 21.3] | 0.618 |
| Assay-valid serum among serum-available patients | Insurance type | Other | 5 (3.2%) | 0 (0.0%) | 0.567 |
| Assay-valid serum among serum-available patients | Insurance type | Urban-rural resident insurance | 49 (31.8%) | 2 (33.3%) | 0.567 |
| Assay-valid serum among serum-available patients | Insurance type | Urban employee insurance | 75 (48.7%) | 2 (33.3%) | 0.567 |
| Assay-valid serum among serum-available patients | Insurance type | Self-pay | 20 (13.0%) | 2 (33.3%) | 0.567 |
| Assay-valid serum among serum-available patients | Insurance type | Commercial supplemental | 5 (3.2%) | 0 (0.0%) | 0.567 |
| Complete four-marker phenotype among assay-valid samples | Age, years |  | 43.5 [37.0, 51.8] | 39.0 [30.8, 57.8] | 0.628 |
| Complete four-marker phenotype among assay-valid samples | Sex | Female | 62 (42.5%) | 2 (25.0%) | 0.470 |
| Complete four-marker phenotype among assay-valid samples | Sex | Male | 84 (57.5%) | 6 (75.0%) | 0.470 |
| Complete four-marker phenotype among assay-valid samples | BMI, kg/m2 |  | 24.9 [22.0, 26.6] | 22.8 [21.5, 23.7] | 0.085 |
| Complete four-marker phenotype among assay-valid samples | Symptom duration, days |  | 39.0 [20.0, 79.5] | 65.0 [50.0, 133.8] | 0.087 |
| Complete four-marker phenotype among assay-valid samples | Baseline leg-pain VAS |  | 6.8 [5.6, 7.8] | 6.2 [5.0, 7.4] | 0.541 |
| Complete four-marker phenotype among assay-valid samples | Baseline ODI |  | 67.1 [58.9, 73.8] | 65.8 [55.6, 69.3] | 0.499 |
| Complete four-marker phenotype among assay-valid samples | Herniation morphology | Extrusion | 71 (48.6%) | 2 (25.0%) | 0.208 |
| Complete four-marker phenotype among assay-valid samples | Herniation morphology | Protrusion | 50 (34.2%) | 3 (37.5%) | 0.208 |
| Complete four-marker phenotype among assay-valid samples | Herniation morphology | Sequestration | 25 (17.1%) | 3 (37.5%) | 0.208 |
| Complete four-marker phenotype among assay-valid samples | Baseline herniation volume, cm3 |  | 0.4 [0.2, 0.6] | 0.3 [0.3, 0.7] | 0.961 |
| Complete four-marker phenotype among assay-valid samples | Initial treatment plan | Surgery planning | 15 (10.3%) | 1 (12.5%) | 1.000 |
| Complete four-marker phenotype among assay-valid samples | Initial treatment plan | Conservative | 94 (64.4%) | 5 (62.5%) | 1.000 |
| Complete four-marker phenotype among assay-valid samples | Initial treatment plan | Nerve root block/injection | 37 (25.3%) | 2 (25.0%) | 1.000 |
| Complete four-marker phenotype among assay-valid samples | Distance to hospital, km |  | 14.2 [7.8, 32.0] | 14.9 [9.2, 29.2] | 0.890 |
| Complete four-marker phenotype among assay-valid samples | Insurance type | Other | 5 (3.4%) | 0 (0.0%) | 0.820 |
| Complete four-marker phenotype among assay-valid samples | Insurance type | Urban-rural resident insurance | 45 (30.8%) | 4 (50.0%) | 0.820 |
| Complete four-marker phenotype among assay-valid samples | Insurance type | Urban employee insurance | 72 (49.3%) | 3 (37.5%) | 0.820 |
| Complete four-marker phenotype among assay-valid samples | Insurance type | Self-pay | 19 (13.0%) | 1 (12.5%) | 0.820 |
| Complete four-marker phenotype among assay-valid samples | Insurance type | Commercial supplemental | 5 (3.4%) | 0 (0.0%) | 0.820 |
| Four-marker phenotype analyzability in source cohort | Age, years |  | 43.5 [37.0, 51.8] | 46.0 [37.2, 55.0] | 0.132 |
| Four-marker phenotype analyzability in source cohort | Sex | Female | 62 (42.5%) | 53 (34.4%) | 0.189 |
| Four-marker phenotype analyzability in source cohort | Sex | Male | 84 (57.5%) | 101 (65.6%) | 0.189 |
| Four-marker phenotype analyzability in source cohort | BMI, kg/m2 |  | 24.9 [22.0, 26.6] | 24.5 [22.7, 27.4] | 0.460 |
| Four-marker phenotype analyzability in source cohort | Symptom duration, days |  | 39.0 [20.0, 79.5] | 63.5 [27.0, 111.0] | 0.005 |
| Four-marker phenotype analyzability in source cohort | Baseline leg-pain VAS |  | 6.8 [5.6, 7.8] | 6.5 [5.6, 7.5] | 0.572 |
| Four-marker phenotype analyzability in source cohort | Baseline ODI |  | 67.1 [58.9, 73.8] | 66.7 [59.8, 72.5] | 0.583 |
| Four-marker phenotype analyzability in source cohort | Herniation morphology | Extrusion | 71 (48.6%) | 63 (40.9%) | 0.027 |
| Four-marker phenotype analyzability in source cohort | Herniation morphology | Protrusion | 50 (34.2%) | 75 (48.7%) | 0.027 |
| Four-marker phenotype analyzability in source cohort | Herniation morphology | Sequestration | 25 (17.1%) | 16 (10.4%) | 0.027 |
| Four-marker phenotype analyzability in source cohort | Baseline herniation volume, cm3 |  | 0.4 [0.2, 0.6] | 0.3 [0.2, 0.5] | 0.069 |
| Four-marker phenotype analyzability in source cohort | Initial treatment plan | Conservative | 94 (64.4%) | 95 (61.7%) | 0.820 |
| Four-marker phenotype analyzability in source cohort | Initial treatment plan | Surgery planning | 15 (10.3%) | 15 (9.7%) | 0.820 |
| Four-marker phenotype analyzability in source cohort | Initial treatment plan | Nerve root block/injection | 37 (25.3%) | 44 (28.6%) | 0.820 |
| Four-marker phenotype analyzability in source cohort | Distance to hospital, km |  | 14.2 [7.8, 32.0] | 13.7 [8.0, 25.7] | 0.490 |
| Four-marker phenotype analyzability in source cohort | Insurance type | Urban-rural resident insurance | 45 (30.8%) | 51 (33.1%) | 0.779 |
| Four-marker phenotype analyzability in source cohort | Insurance type | Urban employee insurance | 72 (49.3%) | 79 (51.3%) | 0.779 |
| Four-marker phenotype analyzability in source cohort | Insurance type | Other | 5 (3.4%) | 5 (3.2%) | 0.779 |
| Four-marker phenotype analyzability in source cohort | Insurance type | Self-pay | 19 (13.0%) | 17 (11.0%) | 0.779 |
| Four-marker phenotype analyzability in source cohort | Insurance type | Commercial supplemental | 5 (3.4%) | 2 (1.3%) | 0.779 |
| Clinical follow-up availability in source cohort | Age, years |  | 45.0 [37.0, 53.0] | 45.0 [37.2, 53.8] | 0.884 |
| Clinical follow-up availability in source cohort | Sex | Female | 82 (35.7%) | 33 (47.1%) | 0.112 |
| Clinical follow-up availability in source cohort | Sex | Male | 148 (64.3%) | 37 (52.9%) | 0.112 |
| Clinical follow-up availability in source cohort | BMI, kg/m2 |  | 24.6 [22.4, 26.8] | 24.9 [22.8, 26.5] | 0.987 |
| Clinical follow-up availability in source cohort | Symptom duration, days |  | 44.5 [21.0, 97.8] | 61.5 [30.2, 96.8] | 0.121 |
| Clinical follow-up availability in source cohort | Baseline leg-pain VAS |  | 6.9 [5.7, 7.8] | 6.2 [5.3, 7.2] | 0.024 |
| Clinical follow-up availability in source cohort | Baseline ODI |  | 66.8 [59.8, 73.1] | 66.8 [57.0, 73.9] | 0.494 |
| Clinical follow-up availability in source cohort | Herniation morphology | Extrusion | 107 (46.5%) | 27 (38.6%) | 0.029 |
| Clinical follow-up availability in source cohort | Herniation morphology | Protrusion | 87 (37.8%) | 38 (54.3%) | 0.029 |
| Clinical follow-up availability in source cohort | Herniation morphology | Sequestration | 36 (15.7%) | 5 (7.1%) | 0.029 |
| Clinical follow-up availability in source cohort | Baseline herniation volume, cm3 |  | 0.4 [0.2, 0.6] | 0.3 [0.2, 0.5] | 0.020 |
| Clinical follow-up availability in source cohort | Initial treatment plan | Conservative | 140 (60.9%) | 49 (70.0%) | 0.192 |
| Clinical follow-up availability in source cohort | Initial treatment plan | Surgery planning | 22 (9.6%) | 8 (11.4%) | 0.192 |
| Clinical follow-up availability in source cohort | Initial treatment plan | Nerve root block/injection | 68 (29.6%) | 13 (18.6%) | 0.192 |
| Clinical follow-up availability in source cohort | Distance to hospital, km |  | 13.8 [7.9, 26.6] | 14.1 [7.8, 30.1] | 0.855 |
| Clinical follow-up availability in source cohort | Insurance type | Urban-rural resident insurance | 72 (31.3%) | 24 (34.3%) | 0.782 |
| Clinical follow-up availability in source cohort | Insurance type | Urban employee insurance | 118 (51.3%) | 33 (47.1%) | 0.782 |
| Clinical follow-up availability in source cohort | Insurance type | Other | 9 (3.9%) | 1 (1.4%) | 0.782 |
| Clinical follow-up availability in source cohort | Insurance type | Self-pay | 26 (11.3%) | 10 (14.3%) | 0.782 |
| Clinical follow-up availability in source cohort | Insurance type | Commercial supplemental | 5 (2.2%) | 2 (2.9%) | 0.782 |
| Paired-MRI endpoint availability in phenotype cohort | Age, years |  | 42.0 [37.0, 51.0] | 45.0 [39.0, 52.0] | 0.346 |
| Paired-MRI endpoint availability in phenotype cohort | Sex | Female | 50 (42.7%) | 12 (41.4%) | 1.000 |
| Paired-MRI endpoint availability in phenotype cohort | Sex | Male | 67 (57.3%) | 17 (58.6%) | 1.000 |
| Paired-MRI endpoint availability in phenotype cohort | BMI, kg/m2 |  | 24.8 [22.0, 26.3] | 25.9 [22.5, 27.5] | 0.250 |
| Paired-MRI endpoint availability in phenotype cohort | Symptom duration, days |  | 35.0 [18.0, 71.0] | 53.0 [34.0, 90.0] | 0.077 |
| Paired-MRI endpoint availability in phenotype cohort | Baseline leg-pain VAS |  | 6.9 [5.7, 7.9] | 6.5 [4.9, 7.6] | 0.132 |
| Paired-MRI endpoint availability in phenotype cohort | Baseline ODI |  | 67.6 [58.1, 74.3] | 65.9 [61.1, 72.9] | 0.906 |
| Paired-MRI endpoint availability in phenotype cohort | Herniation morphology | Extrusion | 63 (53.8%) | 8 (27.6%) | 0.019 |
| Paired-MRI endpoint availability in phenotype cohort | Herniation morphology | Protrusion | 34 (29.1%) | 16 (55.2%) | 0.019 |
| Paired-MRI endpoint availability in phenotype cohort | Herniation morphology | Sequestration | 20 (17.1%) | 5 (17.2%) | 0.019 |
| Paired-MRI endpoint availability in phenotype cohort | Baseline herniation volume, cm3 |  | 0.4 [0.2, 0.6] | 0.2 [0.2, 0.6] | 0.104 |
| Paired-MRI endpoint availability in phenotype cohort | Initial treatment plan | Surgery planning | 9 (7.7%) | 6 (20.7%) | 0.058 |
| Paired-MRI endpoint availability in phenotype cohort | Initial treatment plan | Conservative | 75 (64.1%) | 19 (65.5%) | 0.058 |
| Paired-MRI endpoint availability in phenotype cohort | Initial treatment plan | Nerve root block/injection | 33 (28.2%) | 4 (13.8%) | 0.058 |
| Paired-MRI endpoint availability in phenotype cohort | Distance to hospital, km |  | 14.3 [7.9, 29.3] | 13.7 [7.5, 33.3] | 0.963 |
| Paired-MRI endpoint availability in phenotype cohort | Insurance type | Other | 4 (3.4%) | 1 (3.4%) | 0.635 |
| Paired-MRI endpoint availability in phenotype cohort | Insurance type | Urban-rural resident insurance | 38 (32.5%) | 7 (24.1%) | 0.635 |
| Paired-MRI endpoint availability in phenotype cohort | Insurance type | Urban employee insurance | 58 (49.6%) | 14 (48.3%) | 0.635 |
| Paired-MRI endpoint availability in phenotype cohort | Insurance type | Self-pay | 13 (11.1%) | 6 (20.7%) | 0.635 |
| Paired-MRI endpoint availability in phenotype cohort | Insurance type | Commercial supplemental | 4 (3.4%) | 1 (3.4%) | 0.635 |
| Persistent-pain endpoint availability in phenotype cohort | Age, years |  | 44.0 [37.0, 51.5] | 41.0 [34.5, 51.5] | 0.608 |
| Persistent-pain endpoint availability in phenotype cohort | Sex | Female | 51 (41.5%) | 11 (47.8%) | 0.736 |
| Persistent-pain endpoint availability in phenotype cohort | Sex | Male | 72 (58.5%) | 12 (52.2%) | 0.736 |
| Persistent-pain endpoint availability in phenotype cohort | BMI, kg/m2 |  | 24.8 [21.9, 26.6] | 25.0 [22.6, 26.3] | 0.697 |
| Persistent-pain endpoint availability in phenotype cohort | Symptom duration, days |  | 39.0 [20.0, 78.0] | 45.0 [18.5, 79.5] | 0.891 |
| Persistent-pain endpoint availability in phenotype cohort | Baseline leg-pain VAS |  | 6.9 [5.7, 8.0] | 6.1 [5.0, 7.0] | 0.024 |
| Persistent-pain endpoint availability in phenotype cohort | Baseline ODI |  | 68.0 [59.0, 73.5] | 65.9 [55.5, 74.0] | 0.533 |
| Persistent-pain endpoint availability in phenotype cohort | Herniation morphology | Extrusion | 61 (49.6%) | 10 (43.5%) | 0.299 |
| Persistent-pain endpoint availability in phenotype cohort | Herniation morphology | Protrusion | 39 (31.7%) | 11 (47.8%) | 0.299 |
| Persistent-pain endpoint availability in phenotype cohort | Herniation morphology | Sequestration | 23 (18.7%) | 2 (8.7%) | 0.299 |
| Persistent-pain endpoint availability in phenotype cohort | Baseline herniation volume, cm3 |  | 0.4 [0.2, 0.6] | 0.2 [0.2, 0.5] | 0.072 |
| Persistent-pain endpoint availability in phenotype cohort | Initial treatment plan | Surgery planning | 10 (8.1%) | 5 (21.7%) | 0.032 |
| Persistent-pain endpoint availability in phenotype cohort | Initial treatment plan | Conservative | 78 (63.4%) | 16 (69.6%) | 0.032 |
| Persistent-pain endpoint availability in phenotype cohort | Initial treatment plan | Nerve root block/injection | 35 (28.5%) | 2 (8.7%) | 0.032 |
| Persistent-pain endpoint availability in phenotype cohort | Distance to hospital, km |  | 15.2 [7.8, 29.5] | 13.9 [7.4, 32.3] | 0.776 |
| Persistent-pain endpoint availability in phenotype cohort | Insurance type | Other | 4 (3.3%) | 1 (4.3%) | 0.405 |
| Persistent-pain endpoint availability in phenotype cohort | Insurance type | Urban-rural resident insurance | 37 (30.1%) | 8 (34.8%) | 0.405 |
| Persistent-pain endpoint availability in phenotype cohort | Insurance type | Urban employee insurance | 63 (51.2%) | 9 (39.1%) | 0.405 |
| Persistent-pain endpoint availability in phenotype cohort | Insurance type | Self-pay | 16 (13.0%) | 3 (13.0%) | 0.405 |
| Persistent-pain endpoint availability in phenotype cohort | Insurance type | Commercial supplemental | 3 (2.4%) | 2 (8.7%) | 0.405 |

**Supplementary Table S5. Selection-weight specifications, distributions, and covariate balance.**

| **Selection process** | **Model N** | **Observed N** | **Predictors** | **Phenotype global P** | **Min** | **P1** | **Median** | **P99** | **Max** | **Truncation** |
| --- | --- | --- | --- | --- | --- | --- | --- | --- | --- | --- |
| Source -> four-marker phenotype analyzable | 300 | 146 | age; sex; BMI; symptom duration; baseline leg-pain VAS; baseline ODI; morphology; baseline herniation volume; initial treatment plan; distance to hospital; insurance type |  | 0.625 | 0.658 | 0.938 | 1.815 | 1.996 | 1st-99th percentile |
| Phenotype cohort -> paired MRI complete | 146 | 117 | age; sex; BMI; symptom duration; baseline leg-pain VAS; baseline ODI; morphology; baseline herniation volume; initial treatment plan; distance to hospital; insurance type; immune phenotype | 0.190 | 0.816 | 0.817 | 0.917 | 1.656 | 7.687 | 1st-99th percentile |
| Phenotype cohort -> persistent-pain endpoint complete | 146 | 123 | age; sex; BMI; symptom duration; baseline leg-pain VAS; baseline ODI; morphology; baseline herniation volume; initial treatment plan; distance to hospital; insurance type; immune phenotype | 0.942 | 0.843 | 0.843 | 0.918 | 2.190 | 2.691 | 1st-99th percentile |
| Combined source -> phenotype -> paired MRI | 300 | 117 | Product of source-to-phenotype and phenotype-to-MRI probabilities |  | 0.533 | 0.571 | 0.837 | 2.051 | 11.819 | 1st-99th percentile |
| Combined source -> phenotype -> clinical outcome | 300 | 123 | Product of source-to-phenotype and phenotype-to-clinical probabilities |  | 0.641 | 0.647 | 0.873 | 2.641 | 2.842 | 1st-99th percentile |

*Maximum absolute standardized mean differences by weighting stage:*

| **Stage** | **Max_abs_SMD_before** | **Max_abs_SMD_after** |
| --- | --- | --- |
| Clinical follow-up stage weight: target phenotype cohort | 0.080 | 0.048 |
| Combined serum+MRI weight: target source cohort | 0.266 | 0.059 |
| Combined serum+clinical weight: target source cohort | 0.208 | 0.063 |
| Paired MRI stage weight: target phenotype cohort | 0.112 | 0.037 |
| Source-to-phenotype weight: target source cohort | 0.153 | 0.034 |

**Supplementary Table S6. Endpoint-specific missingness, clinical attrition, and exploratory-composite completeness.**

| **Endpoint** | **Variable** | **Missing, n** | **Model N** |
| --- | --- | --- | --- |
| MRI regression >=50% | MRI regression >=50% | 0 | 117 |
| MRI regression >=50% | Immune phenotype | 0 | 117 |
| MRI regression >=50% | Age | 0 | 117 |
| MRI regression >=50% | Sex | 0 | 117 |
| MRI regression >=50% | BMI | 0 | 117 |
| MRI regression >=50% | Herniation morphology | 0 | 117 |
| MRI regression >=50% | Baseline herniation volume | 0 | 117 |
| MRI regression >=50% | MRI follow-up interval | 0 | 117 |
| Persistent radicular pain | Persistent radicular pain | 0 | 123 |
| Persistent radicular pain | Immune phenotype | 0 | 123 |
| Persistent radicular pain | Age | 0 | 123 |
| Persistent radicular pain | Sex | 0 | 123 |
| Persistent radicular pain | BMI | 0 | 123 |
| Persistent radicular pain | Baseline leg-pain VAS | 0 | 123 |
| Persistent radicular pain | Motor weakness | 0 | 123 |
| Persistent radicular pain | Herniation morphology | 0 | 123 |

*Persistent-pain endpoint availability by phenotype:*

| **Phenotype** | **Phenotype N** | **Pain endpoint observed, n** | **Pain endpoint missing, n** | **Missing, %** |
| --- | --- | --- | --- | --- |
| Low immune activation | 46 | 39 | 7 | 15.2% |
| Cytokine-dominant | 32 | 27 | 5 | 15.6% |
| Chemokine/MMP-dominant | 68 | 57 | 11 | 16.2% |

*Exploratory composite completeness within the MRI-clinical overlap cohort:*

| **Overlap N** | **Composite complete, n** | **Composite incomplete, n** | **Missing MRI regression, n** | **Missing pain endpoint, n** | **Missing 6-month ODI, n** |
| --- | --- | --- | --- | --- | --- |
| 104 | 96 | 8 | 0 | 0 | 8 |

*Within the N=104 MRI-clinical overlap, the eight participants excluded from the N=96 composite were missing follow-up ODI; primary MRI and persistent-pain model covariates were complete within their endpoint-specific cohorts.*

**Supplementary Table S7. Follow-up treatment trajectories and early surgical conversion.**

| **Variable** | **Overall** | **Low immune activation** | **Cytokine-dominant** | **Chemokine/MMP-dominant** | **P value** |
| --- | --- | --- | --- | --- | --- |
| Initial treatment plan | Conservative: 78 (63.4%); Nerve root block/injection: 35 (28.5%); Surgery planning: 10 (8.1%) | Conservative: 26 (66.7%); Nerve root block/injection: 12 (30.8%); Surgery planning: 1 (2.6%) | Conservative: 15 (55.6%); Nerve root block/injection: 8 (29.6%); Surgery planning: 4 (14.8%) | Conservative: 37 (64.9%); Nerve root block/injection: 15 (26.3%); Surgery planning: 5 (8.8%) | 0.473 |
| Epidural steroid injection within 6 months | No: 89 (72.4%); Yes: 34 (27.6%) | No: 28 (71.8%); Yes: 11 (28.2%) | No: 20 (74.1%); Yes: 7 (25.9%) | No: 41 (71.9%); Yes: 16 (28.1%) | 0.975 |
| Analgesic-use days, 0-6 months | 27 [18, 46] | 30 [18, 42] | 32 [23, 66] | 24 [18, 35] | 0.195 |
| Persistent analgesic use within 6 months | No: 51 (41.5%); Yes: 72 (58.5%) | No: 17 (43.6%); Yes: 22 (56.4%) | No: 7 (25.9%); Yes: 20 (74.1%) | No: 27 (47.4%); Yes: 30 (52.6%) | 0.167 |
| Rehabilitation adherence within 6 months | High: 17 (13.8%); Low: 33 (26.8%); Moderate: 33 (26.8%); None: 38 (30.9%); Unknown: 2 (1.6%) | High: 5 (12.8%); Low: 9 (23.1%); Moderate: 11 (28.2%); None: 13 (33.3%); Unknown: 1 (2.6%) | High: 2 (7.4%); Low: 12 (44.4%); Moderate: 7 (25.9%); None: 6 (22.2%) | High: 10 (17.5%); Low: 12 (21.1%); Moderate: 15 (26.3%); None: 19 (33.3%); Unknown: 1 (1.8%) | 0.571 |
| Surgery within 6 months | No: 112 (91.1%); Yes: 11 (8.9%) | No: 37 (94.9%); Yes: 2 (5.1%) | No: 23 (85.2%); Yes: 4 (14.8%) | No: 52 (91.2%); Yes: 5 (8.8%) | 0.386 |
| Rescue visit within 6 months | No: 85 (69.1%); Yes: 38 (30.9%) | No: 24 (61.5%); Yes: 15 (38.5%) | No: 17 (63.0%); Yes: 10 (37.0%) | No: 44 (77.2%); Yes: 13 (22.8%) | 0.195 |

*Early surgery before evaluable follow-up MRI:*

| **Item** | **Overall** |
| --- | --- |
| Early surgery before evaluable follow-up MRI, n | 8 |
| Timing from admission, median [IQR], days | 59 [38, 70] |
| Baseline VAS leg pain, median [IQR] | 7.7 [7.1, 8.5] |
| Baseline ODI, median [IQR] | 77.5 [66.4, 82.0] |
| Motor weakness, n (%) | 2 (25.0%) |
| Baseline herniation volume, median [IQR], cm3 | 0.60 [0.42, 0.90] |
| **Phenotype** | **N** |
| Cytokine-dominant | 6 |
| Chemokine/MMP-dominant | 2 |

**Supplementary Table S8. Full categorical association and sensitivity models.**

| **Endpoint** | **Model** | **Effect** | **N** | **Events** | **Measure** | **Estimate (95% CI)** | **Pairwise P** | **Global P** |
| --- | --- | --- | --- | --- | --- | --- | --- | --- |
| MRI regression >=50% | Primary adjusted; corrected MRI interval | Cytokine-dominant vs Low | 117 | 70 | OR | 0.76 (0.22 to 2.55) | 0.652 | 0.019 |
| MRI regression >=50% | Primary adjusted; corrected MRI interval | Chemokine/MMP-dominant vs Low | 117 | 70 | OR | 2.99 (1.11 to 8.08) | 0.031 | 0.019 |
| Persistent radicular pain | Primary adjusted | Cytokine-dominant vs Low | 123 | 52 | OR | 2.23 (0.65 to 7.65) | 0.203 | 0.003 |
| Persistent radicular pain | Primary adjusted | Chemokine/MMP-dominant vs Low | 123 | 52 | OR | 0.30 (0.09 to 0.95) | 0.042 | 0.003 |
| MRI regression >=50% | Firth penalized sensitivity | Cytokine-dominant vs Low | 117 | 70 | OR | 0.79 (0.24 to 2.48) | 0.680 |  |
| MRI regression >=50% | Firth penalized sensitivity | Chemokine/MMP-dominant vs Low | 117 | 70 | OR | 2.72 (1.07 to 7.18) | 0.036 |  |
| Persistent radicular pain | Firth penalized sensitivity | Cytokine-dominant vs Low | 123 | 52 | OR | 2.05 (0.64 to 6.79) | 0.224 |  |
| Persistent radicular pain | Firth penalized sensitivity | Chemokine/MMP-dominant vs Low | 123 | 52 | OR | 0.34 (0.11 to 0.98) | 0.047 |  |
| MRI regression >=50% | Modified Poisson RR | Cytokine-dominant vs Low | 117 | 70 | RR | 0.87 (0.52 to 1.45) | 0.583 | 0.025 |
| MRI regression >=50% | Modified Poisson RR | Chemokine/MMP-dominant vs Low | 117 | 70 | RR | 1.41 (1.02 to 1.96) | 0.038 | 0.025 |
| Persistent radicular pain | Modified Poisson RR | Cytokine-dominant vs Low | 123 | 52 | RR | 1.24 (0.81 to 1.89) | 0.320 | 0.004 |
| Persistent radicular pain | Modified Poisson RR | Chemokine/MMP-dominant vs Low | 123 | 52 | RR | 0.56 (0.31 to 1.01) | 0.054 | 0.004 |
| MRI regression >=50% | Flexible MRI follow-up time | Cytokine-dominant vs Low | 117 | 70 | OR | 0.76 (0.23 to 2.58) | 0.663 | 0.024 |
| MRI regression >=50% | Flexible MRI follow-up time | Chemokine/MMP-dominant vs Low | 117 | 70 | OR | 2.93 (1.08 to 7.97) | 0.035 | 0.024 |
| MRI regression >=50% | Age nonlinearity sensitivity | Cytokine-dominant vs Low | 117 | 70 | OR | 0.82 (0.24 to 2.78) | 0.745 | 0.065 |
| MRI regression >=50% | Age nonlinearity sensitivity | Chemokine/MMP-dominant vs Low | 117 | 70 | OR | 2.60 (0.95 to 7.11) | 0.062 | 0.065 |
| MRI regression >=50% | Posterior probability >=0.80 | Cytokine-dominant vs Low | 101 | 61 | OR | 0.60 (0.16 to 2.24) | 0.446 | 0.030 |
| MRI regression >=50% | Posterior probability >=0.80 | Chemokine/MMP-dominant vs Low | 101 | 61 | OR | 2.80 (0.96 to 8.11) | 0.058 | 0.030 |
| Persistent radicular pain | Posterior probability >=0.80 | Cytokine-dominant vs Low | 104 | 43 | OR | 2.51 (0.66 to 9.62) | 0.179 | 0.028 |
| Persistent radicular pain | Posterior probability >=0.80 | Chemokine/MMP-dominant vs Low | 104 | 43 | OR | 0.42 (0.12 to 1.48) | 0.175 | 0.028 |
| Favorable structural outcome (MRI regression >=50%; early surgery=failure) | Surgery-as-failure adjusted | Cytokine-dominant vs Low | 125 | 70 | OR | 0.36 (0.12 to 1.11) | 0.075 | 0.002 |
| Favorable structural outcome (MRI regression >=50%; early surgery=failure) | Surgery-as-failure adjusted | Chemokine/MMP-dominant vs Low | 125 | 70 | OR | 2.23 (0.88 to 5.62) | 0.089 | 0.002 |
| Favorable structural outcome (MRI regression >=50%; early surgery=failure) | Surgery-as-failure + initial treatment plan | Cytokine-dominant vs Low | 125 | 70 | OR | 0.46 (0.14 to 1.48) | 0.191 | 0.002 |
| Favorable structural outcome (MRI regression >=50%; early surgery=failure) | Surgery-as-failure + initial treatment plan | Chemokine/MMP-dominant vs Low | 125 | 70 | OR | 2.79 (1.04 to 7.49) | 0.042 | 0.002 |
| MRI regression >=50% | IPW paired-MRI stage; 1/99 truncation | Cytokine-dominant vs Low | 117 | 70 | OR | 0.78 (0.26 to 2.38) | 0.666 | 0.009 |
| MRI regression >=50% | IPW paired-MRI stage; 1/99 truncation | Chemokine/MMP-dominant vs Low | 117 | 70 | OR | 3.54 (1.26 to 9.93) | 0.017 | 0.009 |
| MRI regression >=50% | Combined serum+MRI IPW; 1/99 truncation | Cytokine-dominant vs Low | 117 | 70 | OR | 0.65 (0.21 to 1.99) | 0.453 | 0.002 |
| MRI regression >=50% | Combined serum+MRI IPW; 1/99 truncation | Chemokine/MMP-dominant vs Low | 117 | 70 | OR | 4.24 (1.45 to 12.41) | 0.008 | 0.002 |
| Persistent radicular pain | IPW clinical follow-up stage; 1/99 truncation | Cytokine-dominant vs Low | 123 | 52 | OR | 2.22 (0.78 to 6.29) | 0.133 | 0.007 |
| Persistent radicular pain | IPW clinical follow-up stage; 1/99 truncation | Chemokine/MMP-dominant vs Low | 123 | 52 | OR | 0.30 (0.08 to 1.10) | 0.069 | 0.007 |
| Persistent radicular pain | Combined serum+clinical IPW; 1/99 truncation | Cytokine-dominant vs Low | 123 | 52 | OR | 2.18 (0.76 to 6.31) | 0.148 | 0.014 |
| Persistent radicular pain | Combined serum+clinical IPW; 1/99 truncation | Chemokine/MMP-dominant vs Low | 123 | 52 | OR | 0.32 (0.08 to 1.27) | 0.106 | 0.014 |
| MRI regression >=50% | Storage-adjusted phenotype clustering | Cytokine-dominant vs Low | 117 | 70 | OR | 1.06 (0.34 to 3.29) | 0.915 | 0.019 |
| MRI regression >=50% | Storage-adjusted phenotype clustering | Chemokine/MMP-dominant vs Low | 117 | 70 | OR | 3.54 (1.28 to 9.84) | 0.015 | 0.019 |
| Persistent radicular pain | Storage-adjusted phenotype clustering | Cytokine-dominant vs Low | 123 | 52 | OR | 2.00 (0.60 to 6.59) | 0.256 | 0.002 |
| Persistent radicular pain | Storage-adjusted phenotype clustering | Chemokine/MMP-dominant vs Low | 123 | 52 | OR | 0.25 (0.07 to 0.85) | 0.026 | 0.002 |
| MRI regression >=50% | Five-marker clustering sensitivity | Cytokine-dominant vs Low | 114 | 67 | OR | 0.69 (0.18 to 2.64) | 0.593 | 0.008 |
| MRI regression >=50% | Five-marker clustering sensitivity | Chemokine/MMP-dominant vs Low | 114 | 67 | OR | 3.46 (1.28 to 9.37) | 0.015 | 0.008 |
| Persistent radicular pain | Five-marker clustering sensitivity | Cytokine-dominant vs Low | 119 | 52 | OR | 1.76 (0.51 to 6.13) | 0.374 | 0.035 |
| Persistent radicular pain | Five-marker clustering sensitivity | Chemokine/MMP-dominant vs Low | 119 | 52 | OR | 0.40 (0.13 to 1.19) | 0.100 | 0.035 |
| MRI regression >=50% | Exclude Cook's D >4/N observations | Cytokine-dominant vs Low | 114 | 70 | OR | 0.63 (0.16 to 2.43) | 0.498 | 0.005 |
| MRI regression >=50% | Exclude Cook's D >4/N observations | Chemokine/MMP-dominant vs Low | 114 | 70 | OR | 3.81 (1.27 to 11.43) | 0.017 | 0.005 |
| Persistent radicular pain | Exclude Cook's D >4/N observations | Cytokine-dominant vs Low | 117 | 47 | OR | 2.56 (0.61 to 10.78) | 0.198 | <0.001 |
| Persistent radicular pain | Exclude Cook's D >4/N observations | Chemokine/MMP-dominant vs Low | 117 | 47 | OR | 0.09 (0.02 to 0.42) | 0.002 | <0.001 |
| Persistent radicular pain | Restricted to no surgery by 6 months | Cytokine-dominant vs Low | 112 | 47 | OR | 2.10 (0.58 to 7.68) | 0.260 | 0.007 |
| Persistent radicular pain | Restricted to no surgery by 6 months | Chemokine/MMP-dominant vs Low | 112 | 47 | OR | 0.31 (0.10 to 1.03) | 0.055 | 0.007 |
| Persistent radicular pain | All missing pain outcomes set to no persistent pain | Cytokine-dominant vs Low | 146 | 52 | OR | 1.78 (0.60 to 5.33) | 0.300 | 0.009 |
| Persistent radicular pain | All missing pain outcomes set to no persistent pain | Chemokine/MMP-dominant vs Low | 146 | 52 | OR | 0.36 (0.12 to 1.05) | 0.062 | 0.009 |
| Persistent radicular pain | All missing pain outcomes set to persistent pain | Cytokine-dominant vs Low | 146 | 75 | OR | 2.24 (0.77 to 6.46) | 0.137 | 0.044 |
| Persistent radicular pain | All missing pain outcomes set to persistent pain | Chemokine/MMP-dominant vs Low | 146 | 75 | OR | 0.65 (0.27 to 1.58) | 0.342 | 0.044 |

**Supplementary Table S9. Adjusted continuous structural and clinical outcomes.**

| **Endpoint** | **Model** | **Effect** | **N** | **Adjusted difference (95% CI)** | **Pairwise P** | **Global P** |
| --- | --- | --- | --- | --- | --- | --- |
| Herniation volume reduction, % | Adjusted continuous MRI outcome; HC3; flexible MRI interval | Cytokine-dominant vs Low | 117 | -10.84 (-23.68 to 2.00) | 0.098 | 0.002 |
| Herniation volume reduction, % | Adjusted continuous MRI outcome; HC3; flexible MRI interval | Chemokine/MMP-dominant vs Low | 117 | 9.88 (0.08 to 19.68) | 0.048 | 0.002 |
| 6-month VAS leg pain | ANCOVA-style; baseline VAS adjusted; HC3 | Cytokine-dominant vs Low | 123 | 0.70 (-0.10 to 1.49) | 0.085 | 0.001 |
| 6-month VAS leg pain | ANCOVA-style; baseline VAS adjusted; HC3 | Chemokine/MMP-dominant vs Low | 123 | -0.64 (-1.34 to 0.06) | 0.073 | 0.001 |
| VAS leg pain improvement | Change score with baseline VAS adjustment; HC3 | Cytokine-dominant vs Low | 123 | -0.70 (-1.49 to 0.10) | 0.085 | 0.001 |
| VAS leg pain improvement | Change score with baseline VAS adjustment; HC3 | Chemokine/MMP-dominant vs Low | 123 | 0.64 (-0.06 to 1.34) | 0.073 | 0.001 |
| 6-month ODI | ANCOVA-style; baseline ODI adjusted; HC3 | Cytokine-dominant vs Low | 115 | 4.66 (-1.42 to 10.75) | 0.133 | 0.001 |
| 6-month ODI | ANCOVA-style; baseline ODI adjusted; HC3 | Chemokine/MMP-dominant vs Low | 115 | -5.92 (-11.46 to -0.37) | 0.036 | 0.001 |
| ODI improvement | Change score with baseline ODI adjustment; HC3 | Cytokine-dominant vs Low | 115 | -4.66 (-10.75 to 1.42) | 0.133 | 0.001 |
| ODI improvement | Change score with baseline ODI adjustment; HC3 | Chemokine/MMP-dominant vs Low | 115 | 5.92 (0.37 to 11.46) | 0.036 | 0.001 |

**Supplementary Table S10. Continuous immune-axis association models.**

| **Endpoint** | **Axis** | **N** | **Events** | **OR (95% CI)** | **P** |
| --- | --- | --- | --- | --- | --- |
| MRI regression >=50% | IL-6/IL-8 cytokine axis | 117 | 70 | 0.81 (0.48 to 1.37) | 0.434 |
| MRI regression >=50% | CCL2/MMP-9 remodeling axis | 117 | 70 | 1.68 (1.03 to 2.76) | 0.040 |
| Persistent radicular pain | IL-6/IL-8 cytokine axis | 123 | 52 | 2.38 (1.34 to 4.21) | 0.003 |
| Persistent radicular pain | CCL2/MMP-9 remodeling axis | 123 | 52 | 0.62 (0.37 to 1.07) | 0.084 |

**Supplementary Table S11. Storage-adjusted and five-marker clustering sensitivity analyses.**

| **Sensitivity** | **Classification agreement** | **ARI** | **Endpoint** | **Chemokine/MMP vs Low** | **P value** | **Global phenotype P** |
| --- | --- | --- | --- | --- | --- | --- |
| Storage-adjusted phenotype clustering | 93.8% | 0.822 | MRI regression >=50% | OR 3.54 (1.28 to 9.84) | 0.015 | 0.019 |
| Storage-adjusted phenotype clustering | 93.8% | 0.822 | Persistent radicular pain | OR 0.25 (0.07 to 0.85) | 0.026 | 0.002 |
| Five-marker clustering sensitivity | 93.0% | 0.805 | MRI regression >=50% | OR 3.46 (1.28 to 9.37) | 0.015 | 0.008 |
| Five-marker clustering sensitivity | 93.0% | 0.805 | Persistent radicular pain | OR 0.40 (0.13 to 1.19) | 0.100 | 0.035 |

*MMP-3 was not included in the primary four-marker definition; it was added only in the five-marker sensitivity analysis to avoid redefining the primary data-driven exposure after outcome evaluation.*

**Supplementary Table S12. Primary model diagnostics and complete coefficients.**

| **Endpoint** | **Diagnostic** | **Value** | **Flag** |
| --- | --- | --- | --- |
| MRI regression >=50% | N / events / non-events | 117 / 70 / 47 |  |
| MRI regression >=50% | Predictor degrees of freedom (excluding intercept) | 9 |  |
| MRI regression >=50% | Events per predictor df | 7.78 | Limited information |
| MRI regression >=50% | Smaller outcome category per predictor df | 5.22 | Limited information |
| MRI regression >=50% | Maximum VIF | 1.90 |  |
| MRI regression >=50% | Cook's D > 4/N, n | 3 | Influence sensitivity performed |
| MRI regression >=50% | Maximum Cook's D | 0.124 |  |
| MRI regression >=50% | Leverage > 2p/N, n | 3 |  |
| MRI regression >=50% | Brier score | 0.181 | Descriptive model-fit metric |
| MRI regression >=50% | Hosmer-Lemeshow P (10 groups) | 0.628 |  |
| MRI regression >=50% | Quadratic nonlinearity test: age | P=0.030 |  |
| MRI regression >=50% | Quadratic nonlinearity test: BMI | P=0.057 |  |
| MRI regression >=50% | Quadratic nonlinearity test: baseline herniation volume | P=0.695 |  |
| MRI regression >=50% | Quadratic nonlinearity test: MRI follow-up interval | P=0.865 |  |
| Persistent radicular pain | N / events / non-events | 123 / 52 / 71 |  |
| Persistent radicular pain | Predictor degrees of freedom (excluding intercept) | 9 |  |
| Persistent radicular pain | Events per predictor df | 5.78 | Limited information |
| Persistent radicular pain | Smaller outcome category per predictor df | 5.78 | Limited information |
| Persistent radicular pain | Maximum VIF | 1.44 |  |
| Persistent radicular pain | Cook's D > 4/N, n | 6 | Influence sensitivity performed |
| Persistent radicular pain | Maximum Cook's D | 0.056 |  |
| Persistent radicular pain | Leverage > 2p/N, n | 2 |  |
| Persistent radicular pain | Brier score | 0.157 | Descriptive model-fit metric |
| Persistent radicular pain | Hosmer-Lemeshow P (10 groups) | 0.027 | Potential lack of fit |
| Persistent radicular pain | Quadratic nonlinearity test: age | P=0.074 |  |
| Persistent radicular pain | Quadratic nonlinearity test: BMI | P=0.842 |  |
| Persistent radicular pain | Quadratic nonlinearity test: baseline leg-pain VAS | P=0.656 |  |

*Complete coefficients:*

| **Endpoint** | **Coefficient** | **OR** | **SE (log OR)** | **z** | **P value** | **95% CI lower** | **95% CI upper** | **CI method** |
| --- | --- | --- | --- | --- | --- | --- | --- | --- |
| MRI regression >=50% | Intercept | 0.045 | 2.455 | -1.264 | 0.206 | 0.000 | 5.517 | Wald |
| MRI regression >=50% | Phenotype: Cytokine-dominant vs Low | 0.757 | 0.619 | -0.450 | 0.652 | 0.225 | 2.547 | Wald |
| MRI regression >=50% | Phenotype: Chemokine/MMP-dominant vs Low | 2.991 | 0.507 | 2.160 | 0.031 | 1.107 | 8.080 | Wald |
| MRI regression >=50% | Age, per year | 0.977 | 0.020 | -1.157 | 0.247 | 0.939 | 1.016 | Wald |
| MRI regression >=50% | Male vs female | 1.464 | 0.453 | 0.843 | 0.399 | 0.603 | 3.555 | Wald |
| MRI regression >=50% | BMI, per kg/m2 | 1.070 | 0.084 | 0.810 | 0.418 | 0.908 | 1.261 | Wald |
| MRI regression >=50% | Extrusion vs protrusion | 3.292 | 0.582 | 2.047 | 0.041 | 1.052 | 10.302 | Wald |
| MRI regression >=50% | Sequestration vs protrusion | 15.865 | 1.098 | 2.516 | 0.012 | 1.843 | 136.589 | Wald |
| MRI regression >=50% | Baseline herniation volume, per cm3 | 1.742 | 1.056 | 0.526 | 0.599 | 0.220 | 13.800 | Wald |
| MRI regression >=50% | MRI follow-up interval, per day | 1.005 | 0.005 | 0.965 | 0.334 | 0.995 | 1.015 | Wald |
| Persistent radicular pain | Intercept | 0.000 | 2.637 | -2.968 | 0.003 | 0.000 | 0.070 | Wald |
| Persistent radicular pain | Phenotype: Cytokine-dominant vs Low | 2.227 | 0.629 | 1.272 | 0.203 | 0.649 | 7.647 | Wald |
| Persistent radicular pain | Phenotype: Chemokine/MMP-dominant vs Low | 0.298 | 0.594 | -2.038 | 0.042 | 0.093 | 0.955 | Wald |
| Persistent radicular pain | Age, per year | 1.015 | 0.019 | 0.751 | 0.452 | 0.977 | 1.053 | Wald |
| Persistent radicular pain | Male vs female | 0.914 | 0.466 | -0.194 | 0.846 | 0.366 | 2.279 | Wald |
| Persistent radicular pain | BMI, per kg/m2 | 1.092 | 0.082 | 1.073 | 0.283 | 0.930 | 1.283 | Wald |
| Persistent radicular pain | Baseline leg-pain VAS, per point | 2.285 | 0.198 | 4.171 | <0.001 | 1.550 | 3.369 | Wald |
| Persistent radicular pain | Motor weakness: yes vs no | 3.089 | 0.623 | 1.810 | 0.070 | 0.911 | 10.477 | Wald |
| Persistent radicular pain | Extrusion vs protrusion | 0.367 | 0.525 | -1.908 | 0.056 | 0.131 | 1.028 | Wald |
| Persistent radicular pain | Sequestration vs protrusion | 0.107 | 0.756 | -2.957 | 0.003 | 0.024 | 0.470 | Wald |

**Supplementary Table S13. MRI measurement methods, reader-disagreement records, and archived inter-reader reliability summary.**

| **Recorded volume method** | **N** | **Percent** |
| --- | --- | --- |
| Ellipsoid approximation | 59 | 50.4% |
| Semi-automated volumetry | 33 | 28.2% |
| Reader consensus | 25 | 21.4% |

*Reader-disagreement flag:*

| **Reader disagreement flag** | **N** | **Percent** |
| --- | --- | --- |
| No disagreement flag | 107 | 91.5% |
| Disagreement resolved by consensus | 10 | 8.5% |

Archived inter-reader reliability summary:

| Measure | Statistic | Estimate | 95% CI |
| --- | --- | --- | --- |
| Morphology | Kappa | 0.818 | 0.738 to 0.894 |
| Modic type | Kappa | 0.706 | 0.607 to 0.798 |
| Herniation zone | Kappa | 0.788 | 0.707 to 0.861 |
| Nerve root compression | Weighted kappa | 0.861 | 0.809 to 0.904 |
| Pfirrmann grade | Weighted kappa | 0.804 | 0.718 to 0.872 |
| Regression category | Weighted kappa | 0.876 | 0.785 to 0.938 |
| Baseline herniation volume | ICC | 0.956 | 0.936 to 0.971 |
| Follow-up herniation volume | ICC | 0.942 | 0.908 to 0.968 |
| Regression percent | ICC | 0.834 | 0.783 to 0.873 |

Note: Reliability estimates are reproduced from the archived reader-agreement summary supplied for source verification. The archived records did not retain the reliability sample size, reader training or blinding metadata, exact ICC formulation, intra-reader repeat data, segmentation software/boundary protocol, handling rules for migrated or sequestered fragments, same-platform status, scanner field strength, sequence parameters, or slice thickness. These statistics address inter-reader agreement and do not establish scanner/platform agreement or full technical reproducibility of the volumetric workflow.

**Supplementary Figures**

**Supplementary Figure S1. Analytic availability across nested cohorts.**

Bars show unavailable or incomplete observations at the serum, assay-validity, phenotype, paired-MRI, clinical-follow-up, endpoint, and exploratory-composite stages. Denominators are displayed with each bar. Primary adjusted-model covariates were complete within their endpoint-specific cohorts.

**Supplementary Figure S2. ELISA marker distributions by serum immune phenotype.**

Boxplots show median and interquartile range with individual observations on the ln(value+1) scale. MMP-3 is shown as a secondary marker and was not part of the primary four-marker clustering input.

**Supplementary Figure S3. Covariate balance before and after inverse-probability weighting.**

Absolute standardized mean differences are shown before and after weighting for the source-to-phenotype, phenotype-to-MRI, and phenotype-to-persistent-pain selection models. The dashed line marks an absolute standardized mean difference of 0.10.

**Supplementary Figure S4. Sensitivity analyses for MRI-defined regression.**

Effect estimates for the chemokine/MMP-dominant versus low-activation contrast across primary and sensitivity analyses. OR and RR estimates are identified by plotting symbol and should not be numerically equated. Error bars are 95% confidence intervals.

**Supplementary Figure S5. Sensitivity analyses for persistent radicular pain.**

Effect estimates for the chemokine/MMP-dominant versus low-activation contrast across primary, weighting, classification, surgery-restriction, influence, and extreme-case attrition analyses. Error bars are 95% confidence intervals.
